# Attention Enhances the Retrieval and Stability of Visuospatial and Olfactory Representations in the Dorsal Hippocampus

**DOI:** 10.1371/journal.pbio.1000140

**Published:** 2009-06-30

**Authors:** Isabel A. Muzzio, Liat Levita, Jayant Kulkarni, Joseph Monaco, Clifford Kentros, Matthew Stead, Larry F. Abbott, Eric R. Kandel

**Affiliations:** 1 Department of Neuroscience, Columbia University, College of Physicians and Surgeons, New York, New York, United States of America; 2 Psychology Department, University of Oregon, Eugene, Oregon, United States of America; 3 Mayo Clinic, Rochester, Minnesota, United States of America; 4 Howard Hughes Medical Institute, Columbia University, New York, New York, United States of America; NIMH-NIH, United States of America

## Abstract

A key question in the analysis of hippocampal memory relates to how attention modulates the encoding and long-term retrieval of spatial and nonspatial representations in this region. To address this question, we recorded from single cells over a period of 5 days in the CA1 region of the dorsal hippocampus while mice acquired one of two goal-oriented tasks. These tasks required the animals to find a hidden food reward by attending to either the visuospatial environment or a particular odor presented in shifting spatial locations. Attention to the visuospatial environment increased the stability of visuospatial representations and phase locking to gamma oscillations—a form of neuronal synchronization thought to underlie the attentional mechanism necessary for processing task-relevant information. Attention to a spatially shifting olfactory cue compromised the stability of place fields and increased the stability of reward-associated odor representations, which were most consistently retrieved during periods of sniffing and digging when animals were restricted to the cup locations. Together, these results suggest that attention selectively modulates the encoding and retrieval of hippocampal representations by enhancing physiological responses to task-relevant information.

## Abstract 2

Attention enhances the encoding and retrieval of olfactory and visuospatial representations by modulating place field stability, firing rate, and neuronal synchronization of pyramidal cells in the hippocampus.

## Author Summary

Attention modulates the encoding and retrieval of memories, but the physiological basis of this interaction has largely been unexplored. The formation of memories which depend on the hippocampus involves the conscious recall of events that occur in specific spatial contexts, a form of memory known as episodic. To investigate the physiological consequences of the interaction between attention and memory in the hippocampus, we recorded single-cell activity and local field potentials — the local rhythmic oscillatory activity of neurons — from the same cells over several days while animals learned one of two goal-oriented tasks. In the visuospatial version of the task, mice had to associate a specific spatial location with a reward, independent of an odor cue. In the nonspatial, olfactory version, mice had to associate a specific odor with the food reward, independent of spatial location. We found that, during periods of navigation, only neurons in the visuospatially trained animals displayed long-term stable representations of space, and neuronal synchronization to so-called gamma oscillations, a mechanism of signal amplification that has been proposed to underlie attentional processes. Conversely, when animals were sniffing the odors in fixed spatial locations, only neurons in the olfactory-trained group displayed a stable increase in firing rate in response to the reward-associated odor. Our data suggest that attention modulates what is encoded and retrieved by hippocampal cells and that neuronal synchronization to gamma oscillations may underlie the mechanism whereby attention leads to stable spatial memory retrieval during navigation.

## Introduction

Evidence from both human and animal research suggests that the hippocampus is involved in processing episodic memory [1],[2], a form of memory for sequential events that requires attention, both for optimal encoding and subsequent retrieval [3],[4]. Even though the involvement of the hippocampus in this type of memory has been well documented using a variety of approaches [5], the manner in which attentional processes modulate memory consolidation is not well understood. Specifically, it is not known how attention to different environmental cues affects the long-term retrieval of information at the single-neuron and network levels.

One of the characteristics of hippocampal cells that supports the role of this region in episodic memory is that these neurons fire in response to particular events or episodes, for example, the start and end point of a particular trajectory through space [6]–[8]. These responses rely on the property of hippocampal cells to fire in particular locations as animals move in the environment—the cell's place field [9]. The stable retrieval of place fields, whereby the same cell fires in the same circumscribed location when the animal is re-introduced to the same environment, requires the same biochemical cascades that are necessary for memory consolidation [10]–[12]. This is consistent with the idea that place field stability is a neural process underlying long-term episodic spatial memory. At present, however, very few studies have investigated the behavioral and physiological variables that affect the long-term stability of place fields because of the difficulty associated with obtaining long-term recordings from the same cells over a period of several days. Moreover, the few studies that have addressed this issue have only focused on the retrieval of spatial representations [10]–[14].

The hippocampus, however, not only encodes spatial information but also time relationships, as well as other types of sensory information such as olfactory and auditory cues [15]–[20]. Most of these nonspatial aspects of the environment are represented at the physiological level by changes in firing rate [20],[21]. Importantly, the changes in firing rate as well as the re-mapping of place fields are controlled by task contingencies [19]–[22], a process that appears to be modulated by attention [22]. Yet, it is still not clear whether nonspatial representations could be stable in the long term or whether attention to different task contingencies could differentially affect the long-term retrieval of spatial and nonspatial representations. Importantly, task contingencies also affect the activity of neuronal ensembles by modulating the synchronization of local oscillation patterns. However, processing of hippocampal information at the network level has been primarily studied in the short-term or after task acquisition [23]–[25], which has not allowed the evaluation of how the network synchronicity patterns change during the learning process over time (but see [26]).

Having refined the methods for recording from the same cells for several days, we previously investigated the effect of the behavioral context on the retrieval of spatial representations [27]. We recorded neural activity in the dorsal hippocampus in different groups of mice performing tasks that varied systematically with the degree of behavioral demands, ranging from no task demands (free exploration) to executing an active avoidance goal-oriented spatial task. We found that the degree of long-term stability of the place fields correlated with the degree to which the animal assigned behavioral significance to the visuospatial landmarks in the environment. Place fields were stable only when the task required the mice to attend to the spatial layout of the environment (see also [28]). Even though these results strongly suggested a role for attention in the stabilization of spatial hippocampal representations, these findings raised the following three questions: First, can the stability of visuospatial representations be achieved by a general state of arousal or does this process require attention to the visuospatial environment? Second, does attention to nonspatial cues lead to the emergence and/or long-term stabilization of task-dependent, nonspatial representations? Finally, when animals learn to attend to a sensory dimension that increases place field stability, what physiological changes correlate with this attention process at the network level?

To address these questions, we recorded single-unit activity and the local field potential from pyramidal neurons in the dorsal hippocampus over five consecutive days while animals acquired one of two goal-oriented tasks that required attention to either fixed visuospatial or spatially shifting olfactory cues to retrieve a hidden food reward. We determined how these different task contingencies affected the retrieval of hippocampal representations by analyzing spike activity during periods of active exploration and periods of sniffing and digging, when animals were confined to a particular spatial location in close proximity to the odors. We found that, during navigation, the stabilization of the place field map required attention to the visuospatial environment. The increase in place field stability in the visuospatial group was concomitant with an increase in spike phase locking to gamma oscillations, a putative mechanism of attention thought to underlie signal amplification [29],[30]. Attention to a spatially shifting olfactory cue led to the emergence of task-dependent representations that were most consistently retrieved during periods of sniffing and digging when the animals were restricted to the cup locations. All together, these findings indicate that in the hippocampus, attention modulates encoding and retrieval of spatial and task-relevant, nonspatial representations.

## Results

### Visuospatial and Olfactory Goal-Oriented Tasks

**Figure 1:**
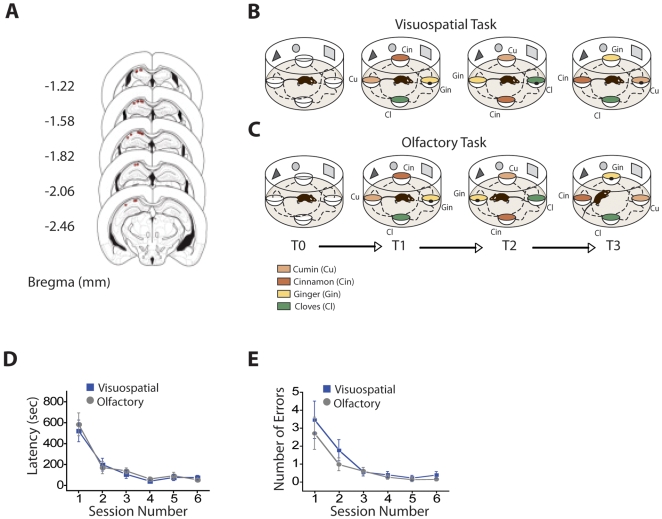
Experimental design and task acquisition. (A) Recording location. Schematic diagram of tetrode placements in the left dorsal hippocampus CA1 pyramidal cell layer (red circles). (B and C) Goal-oriented tasks. Two groups of mice were trained to find a hidden food reward buried inside one of four cups, which were filled with odor-scented bedding. (B) In the visuospatial task, the location of the reward remained fixed throughout training but the scented bedding covering the reward changed from trial to trial. (C) In the olfactory task, the location of the reward shifted from trial to trial in a pseudo-random fashion, but the scented bedding covering the reward remained constant. Odors used were cumin (cu), cinnamon (cinn), cloves (cl), and ginger (gin); the black dot placed on top of one of the cups represents the hidden food reward. (D and E) Task acquisition was equivalent in both groups as illustrated by the similar reduction in (D) latency to find the reward and (E) number of errors [latency: *F*(5,88) = 22.71, *p*<0.001; errors: *F*(5,88) = 12.48, *p*<0.001], with no significant difference between the two groups in the rate of acquisition [latency: group: *F*(1,88) = 0.07, *p* = 0.78; interaction: *F*(5,88) = 1.07, *p* = 0.38; errors: group: *F*(1,84) = 0.25, *p* = 0.63; interaction: *F*(5,84) = 0.78, *p* = 0.57]. Blue: visuospatial group: *n* = 12; Gray: olfactory group: *n* = 11; Line plots show session mean±SEM.

We recorded unit activity from CA1 pyramidal neurons of the dorsal hippocampus (Figure 1A) in two groups of mice that were trained in either a visuospatial or an olfactory goal-oriented navigational task. In the visuospatial task, mice had to attend to the visuospatial cues in the environment to find a particular location where the reward was placed, while ignoring the odor that covered the reward in each trial (Figure 1B). In the olfactory task, mice had to attend to a specific odor associated with the reward, while ignoring the spatial location where that odor and reward were placed in each trial (Figure 1C). In each of these tasks, the food reward consisted of small pieces of cereal hidden inside one of the cups under a layer of scented bedding (see Materials and Methods). Animals were trained in these tasks for three consecutive days receiving two four-trial sessions per day. On the fourth day, a series of control trials tested how manipulations of the task-relevant cue affected task performance and the firing characteristics of the cells we recorded throughout training (Figure S1A). We found that in both tasks, mice reached asymptotic levels of learning after three to four sessions (day 2 of training), with no significant difference between the two groups in the rate of task acquisition, as measured by the reduction in both latency and errors made to find the reward (Figure 1D and 1E, respectively).

We also examined locomotor parameters and other task-related behaviors to rule out the possibility that these variables might have differentially affected the overall time and/or speed of movement of animals in either the visuospatial or olfactory groups. We found that mice in both groups exhibited equivalent levels of locomotor activity and digging time across all training trials [olfactory group: *n* = 11 mice; average speed (cm/s) = 5.96±0.38, path length (cm) = 4996.28±386; average digging time per session (s) = 1222±80; visuospatial group: *n* = 12; average speed (cm/s) = 6.08±0.23, path length (cm) = 5224.13±248; average digging time per session (s) = 1224±126, see also Figure S1C and S1D for data showing digging time during training and Figure S2 for path trail examples]. These results support the idea that the changes observed at the neural level between the two groups were not the result of differences in the rate of task acquisition, procedural demands, exploratory activity, or levels of arousal between the two groups.

### Place Field Stability during Navigation Requires Attention to Space

To assess the effect of our goal-oriented tasks on the retrieval of spatial representations, we first measured the short- and long-term stability of place fields in the visuospatial and olfactory groups (see Materials and Methods). The stability of place fields was measured over four consecutive days while animals acquired the tasks described above during periods of locomotion when hippocampal cells are maximally active (minimum locomotion speed threshold: 2 cm/s) [31]. During this 4-d period we recorded from the same neurons in the dorsal hippocampus (visuospatial group, day 1: 12 animals, 58 cells; day 4: 6 animals, 24 cells. Olfactory group, day 1: 11 animals, 64 cells; day 4: 8 animals, 41 cells). We predicted that if a general state of arousal, rather than selective attention to space, is sufficient to produce place field stability, then both the visuospatial and olfactory groups should display stable place fields. Conversely, if attention to space is required, then only animals in the visuospatial group that attended to the visuospatial environment should display place field stability.

**Figure 2:**
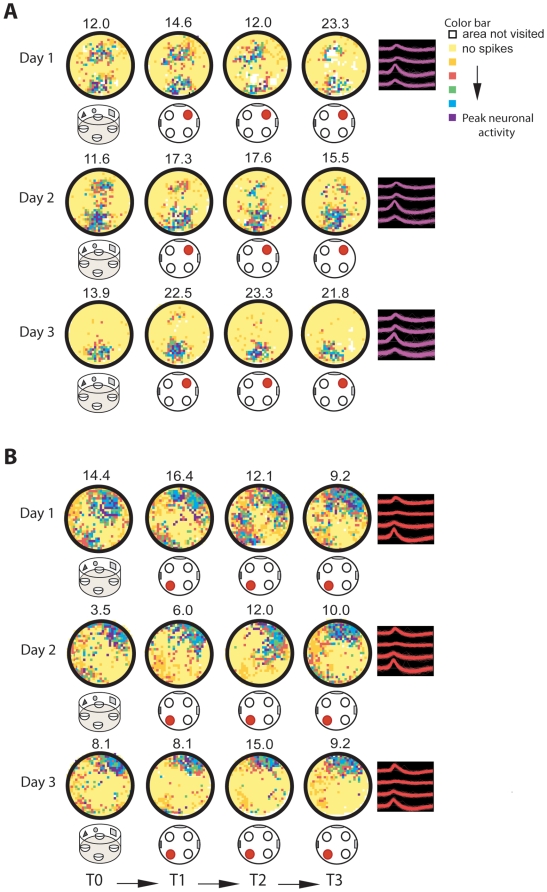
Attention to the visuospatial environment enhances place field stability. Color-coded rate maps showing firing activity of two single CA1 pyramidal cells over three sequential days in animals trained in the visuospatial group. Below each rate map, a cartoon of the arena marks the position of the reward with a red circle. The four waveforms on the right represent a tetrode recording from a single cell. The constancy of the waveforms throughout the three days of training demonstrates recording stability. On day 1, both cells (A and B) displayed unstable and disorganized place fields. As animals learned to attend to the visuospatial environment, the stability and organization of the fields was significantly enhanced. This effect was evident during training and probe trials (T0). Color map indicates neuronal level of activity. White pixels are regions that the animal never visited. Yellow pixels are regions the animal visited but the cell never fired. Orange, red, green, blue, and purple pixels progressively encode higher firing rates that are auto-scaled relative to the peak firing frequency (shown above each rate map).

**Figure 3:**
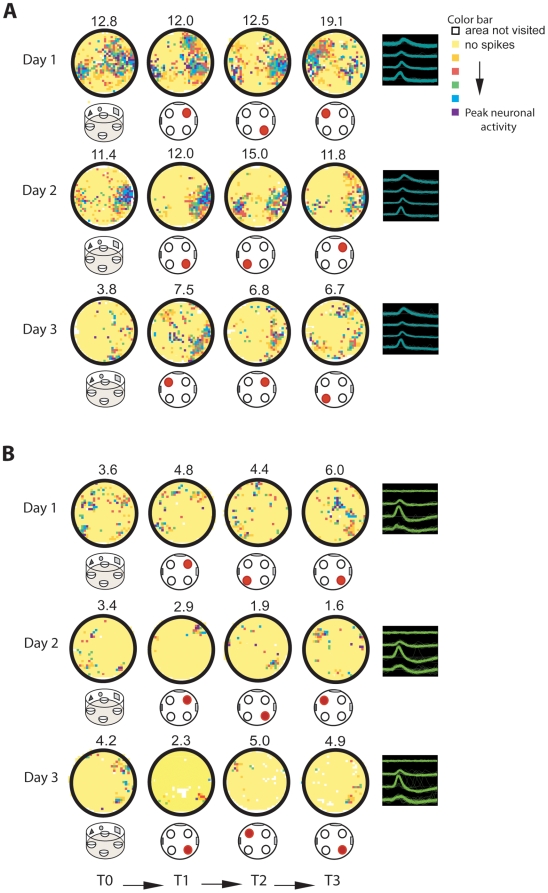
Attention to a spatially shifting olfactory cue compromises place field stability. Color-coded rate maps showing firing activity of CA1 pyramidal cells over three sequential days in animals trained in the olfactory group. Color maps, cartoon notations, and waveforms represent the same parameters shown in Figure 2. (A) Olfactory group, cell type 1: A general characteristic of this cell type was the emergence of multiple fields with one often locked to the reward-associated odor. These cells became highly disorganized with successive trials. This effect was evident during training as well as during the probe trial (T0). (B) Olfactory group, cell type 2: In these cells location-specific firing disintegrated quickly. As training progressed, the firing fields of these cells coincided with the location of the reward-associated odor.

**Figure 4:**
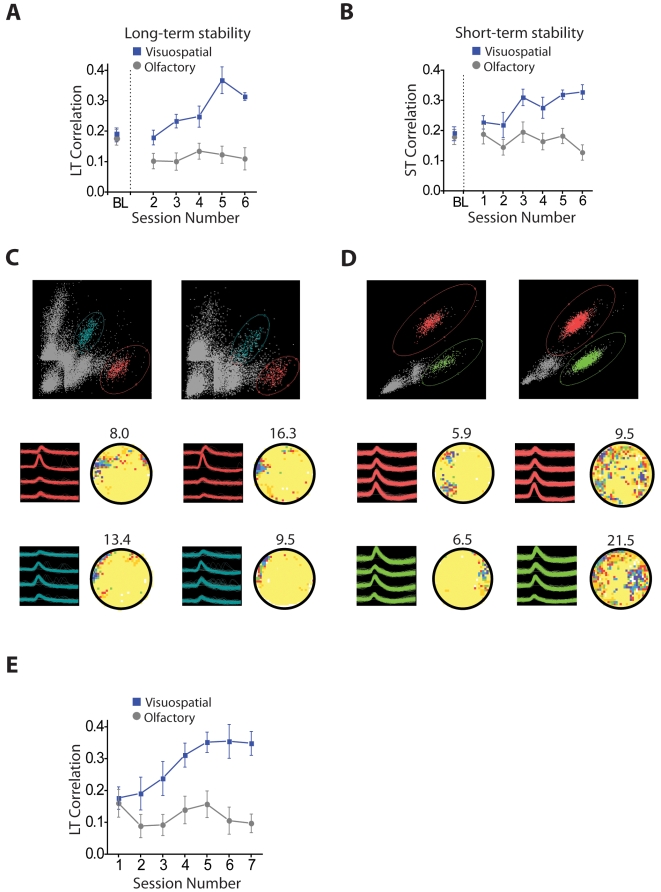
During navigation, attention modulates the stability of spatial representations during task performance and free exploration. (A) Long-term place field stability was analyzed by correlating neuronal activity between the first training trials of each session. Before task acquisition, both groups, visuospatial and olfactory, showed similar levels of stability. After animals learned to attend to the relevant rule, stability was significantly enhanced in animals in the visuospatial group, remaining low after only one training session in the olfactory group. Groups: *F*(1,63) = 26.93, *p*<0.001; session: *F*(4, 63) = 5.49, *p*<0.001; interaction: *F*(4,63) = 3.15, *p*<0.02, groups were different on sessions 2, 3, 4, 5, and 6, *p*<0.05. (B) Short-term stability was calculated by averaging the correlation values between training trials in each session. Learning to attend to the relevant percept significantly enhanced the short-term stability in the visuospatial group and reduced it in the olfactory group [groups: *F*(1,83) = 15.20, *p*<0.001; session: *F*(5,83) = 4.22, *p*<0.002, groups were different on sessions 2, 3, 4, 5, and 6, *p*<0.05; interaction: *F*(5,83) = 3.34, *p*<0.008]. (C and D) Examples of cluster projections and rate maps of cells recorded from animals in the visuospatial (C) and olfactory (D) groups on days 1–3 demonstrating recording stability. Color maps and waveforms represent the same parameters shown in Figure 2. (E) Correlation coefficients calculated during the probe trials (T0) in sequential sessions revealed enhanced long-term place field stability in animals trained in the visuospatial, but not the olfactory group [group: *F*(1,97) = 22.26, *p*<0.001; session: *F*(6,97) = 2.49, *p*<0.03; interaction: *F*(6,97) = 2.58, *p*<0.03]. Post hoc analysis showed that the groups were significantly different on session 3, 4, 5, 6, and 7 (*p*<0.02), but not on session 1 or 2 before the animals learned the task (*p*>0.05). Line plots show session mean±SEM. BL, baseline.

Before training commenced, place fields in both groups displayed very similar low levels of stability during free exploration of the environment (visuospatial group: *r* = 0.18±0.03; olfactory group: *r* = 0.16±0.04, *F*(1,22) = 0.089, *p* = 0.768). These values were comparable to those previously reported in mice in the open field under no task contingencies [27]. Training in the visuospatial task produced a gradual and significant increase in both short- and long-term place field stability that was maximal after animals reached asymptotic levels of task-performance (Figures 2A, 2B) [before training (Day 1, T0): *r* = 0.18±0.03; end of training (Day 3, session 6): short-term *r* = 0.33±0.02, long-term *r* = 0.35±0.02]. In contrast, the overall place field stability of cells from animals trained in the olfactory task was significantly reduced at the end of training. In this group, the long-term stability degraded within one training session; however, the decrement in short-term stability occurred gradually over the course of training (Figures 3A, 3B, 4A, and 4B) [before training (Day 1, T0): *r* = 0.16±0.04; end of training (Day 3, session 6): long-term *r* = 0.11±0.04; short-term *r* = 0.13±0.03]. In this group, lack of place field stability was observed in the great majority of the cells (83%, 41 cells).

In the visuospatial, but not the olfactory, group the changes in stability were concomitant with an enhancement in both coherence—a parameter that reflects the degree of organization of the place field—and information content—a parameter that evaluates how well the firing of each cell predicts the animal's location [22] (coherence: session 6, visuospatial = 0.41±0.04, olfactory = 0.25±0.04; group, F(1,84) = 7.58, *p*<0.02; session: *F*(5,84) = 2.59, *p*<0.04. interaction: *F*(5,84) = 2.74, *p*<0.04; groups were significantly different in sessions 3, 4, and 6, *p*<0.05. information content: session 6, visuospatial = 2.07±0.11, olfactory = 1.33±0.13; group and session not significant; interaction, *F*(5,84) = 3.58, *p*<0.005; groups were significantly different in sessions 5 and 6, *p*<0.05, unpublished data). Other parameters such as field size and average firing rate did not display differences between the groups. While field-size displayed a moderate decrease across sessions during training in both groups (*p*<0.03), the average firing rate was constant in both conditions [visuospatial (spikes/s): 1.02±0.11; olfactory (spikes/s): 1.13 ± –0.11, *p* = 0.307, see Figure S3].

We then asked if the changes we observed during training were merely the result of different cognitive demands during task performance or they could generalize to the training context when animals were not performing the task. To address this question, we examined the place fields during the probe trials (T0), where animals freely explored the experimental arena before the start of each training session in the absence of task contingencies (Figure 1B and 1C). We found that by the last day of training, cells recorded from animals in the visuospatial group showed a 100% increase in place field stability during these trials, whereas cells from animals in the olfactory group displayed a decrease in place field stability of nearly 40% (Figure 4E) (visuospatial group: session 1, *r* = 0.17±0.03; session 6, *r* = 0.34±0.03; olfactory group: session 1, *r* = 0.16±0.03; session 6, *r* = 0.10±0.04). This effect was concomitant with a significant difference between the groups in information content [group: *F*(1,98) = 4.51, *p*<0.05; interaction: *F*(6, 98) = 2.58, *p*<0.03, groups were different on sessions 5, 6, and 7, *p*<0.05; unpublished data). Other spatial parameters, such as coherence, and nonspatial parameters, such as field size and overall firing rate, were not significantly different between the visuospatial and olfactory groups. Together, these data show that learning to attend to a stable visuospatial environment is critical for successful retrieval of spatial representations and this effect can generalize to situations where animals are not performing task contingencies.

### Reward-Associated Odor Representations in the Dorsal Hippocampus Are Stable during Periods When the Animals Are Processing Odors at Fixed Spatial Locations

During periods of navigation, the place field instability observed in the great majority of cells recorded in the olfactory group (83%, 41 cells) was the result not only of the unstable retrieval of the place fields, but also the emergence of task-dependent representations. At the end of training (day 3, session 6), we could classify cells recorded from animals in the olfactory group into two groups: (1) neurons that displayed reward-associated odor activity (56%, 23 cells) and (2) neurons that displayed unstable and disorganized place fields (44%, 18 cells, see Materials and Methods). The reward-associated odor phenotype was observed in cells that developed multiple disorganized fields with one coinciding with the rewarded-odor location (Figure 3A, session 6, 17 cells) or in cells that displayed one field that shifted and re-mapped according to the location of the rewarded odor (Figure 3B, session 6, 6 cells).

To test whether representations locked to the reward-associated odor were stable in the short- and long term, we first examined the stability of the firing fields during periods of locomotion (minimum speed threshold: 2 cm/s) using the position of the reward-associated odor as the reference frame for the analysis (see Materials and Methods, Rotational Analysis section). These values were compared to those obtained in the visuospatial condition using the visuospatial reference frame. We found that within each session (short-term stability measure), the reward-associated odor representations displayed high levels of stability that were comparable to the values obtained for place fields in the visuospatial group. A cell that fired in the position of the reward-associated odor on trial one was likely to retrieve the same representation on subsequent trials within the same session [session 6, intertrial interval (ITI) 2 min, olfactory: *r* = 0.31±0.05; visuospatial: *r* = 0.32±0.03; *F*(1,11) = 0.21, *p* = 0.65]. However, when we examined the stability over the long term, we found that odor representations in the olfactory group displayed lower levels of stability in comparison to the spatial representations recorded in animals in the visuospatial group [session 6, inter-session interval 7–8 or 12–14 h, olfactory: *r* = 0.20±0.02; visuospatial: *r* = 0.30±0.02; *F*(1,9) = 7.52, *p*<0.03], i.e., in the olfactory group, the same cell tended to switch between different representational phenotypes between sessions (Figure S4). This observation differed from the characteristics of spatial representations in the visuospatial group, which showed both short- and long-term stability. However, it is possible that during periods of navigation, olfactory representations were masked by the emergence of spatial representations that were formed as result of having specific odors present in particular spatial locations within each trial. Moreover, since olfactory cues diffuse in space across a gradient that becomes weaker with distance from the cups, odor representations could have been more prominent when animals were in close proximity to the cups. Therefore, to determine if olfactory representations were stable in the long term, it was also necessary to examine neural responses during times of digging and sniffing, when the animals were experiencing the odors inside the cups, in fixed spatial locations.

To this end, we restricted the analysis of neuronal activity to the areas where the cups were located during periods when the animals were not walking, e.g., only movement speeds lower than 2 cm/s were included in this analysis. We used this low threshold speed to capture slight head movements produced during digging and sniffing inside the cups (each cup was 5 cm in diameter). This analysis was performed only in those animals in which we held the cells throughout training: visuospatial, *n* = 6; olfactory, *n* = 8. We found that the firing activity of hippocampal cells was slightly lower when the animals were sniffing and digging than when they were actively exploring the environment as it has been previously described in the literature [32] The decrease in firing rate during periods of sniffing and digging relative to periods of locomotion was 28±6.9% in the visuospatial group and 23±5.2% in the olfactory group. The difference between the groups was not statistically significant [*F*(1,13) = 0.40, *p* = 0.53].

**Figure 5:**
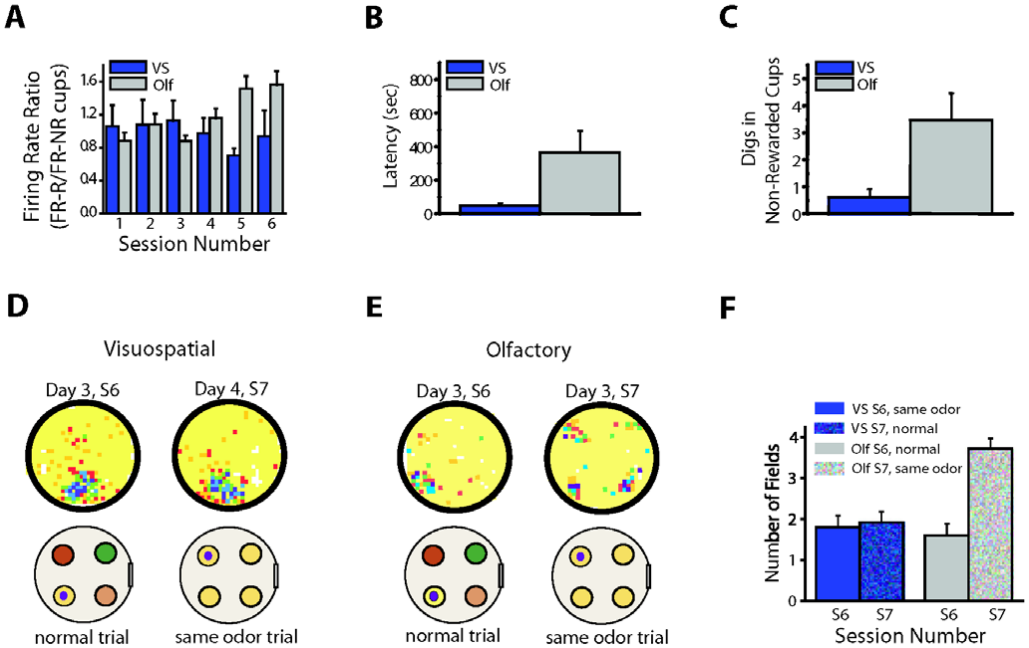
Attention to a task-relevant olfactory cue enhances retrieval of a reward-associated odor. (A) Firing rate responses to the reward-associated odor increased during periods of sniffing and digging only in the olfactory group. (B–F) Same odor trial: During this trial the four cups in the arena contained the same scented bedding, but the reward was hidden in only one of the cups. (B and C) The same odor trial only affected the behavior of olfactory animals as illustrated in the increase in the latency to find the reward (B) and the number of digs in the non-rewarded cups (C). (D and E) Rate maps of animals trained in the visuospatial (D) and olfactory (E) groups during the same odor trial. (D) The place fields of animals in the visuospatial group were not affected. (E) In the olfactory group, however, the fields that coincided with the location of the reward-associated odor during training broke down into four fields locked to the position of the four cups. Cartoon below each rate map indicates the positions of odors and the buried reward (black dot) on each trial. The color map is the same as that shown in Figure 2. (F) Number of fields associated with the four-cup locations during the last training session [day 3, session 6 (S6)] and the same odor trial [day 4, session 7 (S7)] recorded in the visuospatial and olfactory groups. The number of fields significantly increased during the same odor trial in the olfactory group [*t*(6) = −3.48, *p*<0.02], showing no significant difference in the visuospatial group [*t*(6) = 1, *p* = .356]. Histograms show trial mean±SEM. Olf, olfactory; S, session; VS, visuospatial, yellow: ginger, green: cumin, red: cinnamon, pink: cloves.

Despite the small reduction in the overall firing rate during periods of sniffing and digging, we found that cells consistently fired over rewarded and nonrewarded cups, which allowed us to determine if there were firing rate differences between groups when the animals were circumscribed to the cup locations. To this end, we first divided the firing rate over the rewarded cup by the average firing rate over the nonrewarded cups. Values above 1 indicated that the cells fired more strongly in response to the reward-associated odor and reward; values below 1 indicated that the cells fired more strongly in response to the nonrewarded odors in different locations. We found that at the end of training, after animals learned to attend to the relevant percept to find the reward, cells from animals in the olfactory group showed a significant increase in the firing rate ratio in comparison to the cells from animals in the visuospatial group, which did not display significant differences at any point during training (see Figure 5A; mean firing rates on session 6 (spikes/s): visuospatial: 0.94±0.31; olfactory: 1.57±0.17; interaction: *F*(5,64) = 3.38, *p*<0.009). The differences between the groups were significant on session 5 and 6 (*p*<0.05; see also Table S1).

To determine if the cells also responded to the nonrewarded odors, we calculated a ratio for each of the nonrewarded odors by dividing the firing rate in response to each of these odors by the average firing rate of the remaining nonrewarded odors. We found that none of the visuospatial animals displayed increased firing rate in response to a nonrewarded odor. In the olfactory group, only one out of eight animals displayed significantly higher firing rate in response to one (cinnamon) of the three nonrewarded odors [*F*(2,17) = 6.35, *p*<0.02]. In this animal, there were no significant differences between the ratios obtained for the nonrewarded odor (cinnamon) and the rewarded odor (cumin) [*F*(1,10) = 1, *p* = 0.34]. Interestingly, this animal was one of the slowest learners in the olfactory group as indicated by the long latency to find the reward in comparison to other animals in the group (see Table S2), which further supports our idea that learning to attend to task rules is necessary for the proper retrieval of task-relevant information. In summary, these data show that task-relevant odors are consistently retrieved when animals learn to attend to that odor. These results are also consistent with other studies showing that hippocampal changes in firing rate code additional information about the animal's environment [33].

### Reward-Associated Odor Representation Can Be Evoked at Multiple Spatial Locations

The goal of this study was to examine how attention to spatial or nonspatial task contingencies affected hippocampal long-term memory representations. To test whether animals in the olfactory group indeed learned to attend to the nonspatial odor cue to find the reward, we ran a same odor trial during session 7 on day 4. All control trials were conducted only once (see below and Material and Methods). During this trial, the four cups in the arena contained the same scented bedding, with the reward buried in only one of the four cups. In the olfactory group, the odor used during this trial was the one that predicted the reward during training, and the position of the reward location was picked randomly. For animals in the visuospatial group, the odor placed in the four cups was picked randomly, but the position of the reward was the same one used during training. Since the same odor trial disrupted the relationship between the task-relevant cue and the reward, we conducted this trial only once to avoid extinction of the learned association.

If animals in the olfactory group learned to attend to a particular odor to find the reward, their performance would be severely impaired during this trial. In contrast, animals in the visuospatial group should not be affected by this manipulation, because these animals learned to ignore the odors in order to correctly perform the task. Consistent with this idea, we found that animals in the olfactory group showed a significant increase in the latency to find the reward resulting from digging in the nonrewarded cups, whereas animals in the visuospatial condition were not affected by the same odor manipulation (Figure 5B and 5C) [latency to find the reward (s): visuospatial: 53.63±20.27; olfactory: 369.75±139.75, *p*<0.04; number of digs in non-rewarded cups: visuospatial: 0.63±0.24; olfactory: 3.5±1.32, *p*<0.02].

At the neuronal level, the same odor trial also allowed us to assess the nature and manner in which the odor representations were encoded by providing a way to answer the following two questions. First, were these cells responding to the reward-associated odor or the reward itself? Second, were these hippocampal cells responding to the particular location where the rewarded cup was placed or were they responding to the reward-associated odor independent of specific spatial coordinates? If odor representations were co-localized with the position of the reward or the reward-associated odor within a particular spatial location, then, during the same odor trial, these cells would fire only near the cup that contained the reward and not the other cup locations. Conversely, if the cells were responding to the reward-associated odor independently of a single spatial reference frame, they would fire at all four cup locations. To assess these possibilities, we analyzed the firing properties of hippocampal cells during two periods: (1) active exploration, when animals were navigating in the environment, and (2) sniffing and digging at the cup locations. Both these analyses were conducted by comparing the firing activity on the last training session (session 6, day 3) with that recorded during the same odor trial (session 7, day 4).

During periods of exploration, we found that in the olfactory group, the same cells that fired at the location of the reward-associated odor during training fired on top of the four cup locations during the same odor trial (Figure 5E). This was illustrated by the increase in the number of fields in response to the reward-associated odor, which was only observed in the olfactory group during the same odor trial (Figure 5F) (number of fields: visuospatial: day 3, session 6, trial 3 = 1.55±0.2; day 4, session 7, same odor trial = 1.54±0.21; olfactory: day 3, session 6, trial 3 = 1.66±0.14; day 4, session 7, same odor trial = 2.85±0.3). This effect was in sharp contrast to the neural activity of cells in animals in the visuospatial group, where this manipulation had no effect on the location where the cells fired as indicated by the high place field stability (Figure 5D) [visuospatial: *r* = 0.34±0.05; olfactory: *r* = 0.11±0.06; t(11) = 3.20, *p*<0.009].

To further explore the effects of the same odor trial on the firing characteristics of dorsal hippocampal cells, we then examined firing rate activity only during periods of digging and sniffing, when the reward-associated odor representations were more prominent during training (see previous section). As expected, in the visuospatial group, the firing rate responses to the odors were unaffected, showing the same pattern during training and the same odor trial [firing rate ratio (rewarded/non-rewarded odors), session 6: 0.94±0.31; session 7 (same odor trial): 0.86±0.30; *t*(6) = 2.47, *t*(4) = −0.04, *p* = 0.996]. In the olfactory group, however, during the same odor trial, the firing rate was equivalent among the rewarded and the nonrewarded odors, in strong contrast to the firing activity observed during the last training session (day 3 session 6), where the recorded cells fired more strongly in response to the reward-associated odor than to the nonrewarded odors [firing rate ratio (rewarded/non-rewarded odors), session 6: 1.57±0.17; session 7 (same odor trial): 1.07±0.03; unpublished data]. These results indicate that cells in the olfactory group that fired at the four cup locations were not responding to the reward itself, but rather to the odor that had been associated with the reward. Furthermore, these data show that dorsal hippocampal neurons can code nonspatial information at multiple locations, independently of a single spatial reference frame.

### Only Animals Trained in the Visuospatial Task Display Behavioral and Neuronal Responses Locked to the Proximal Visuospatial Landmarks in the Environment

**Figure 6:**
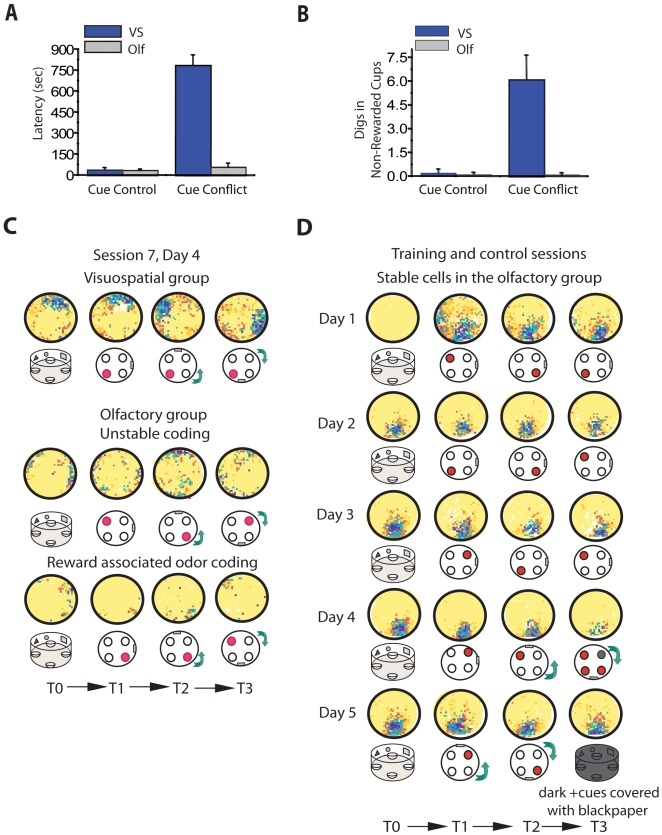
Place fields from animals trained in the visuospatial task are locked to the proximal visuospatial cues in the environment. (A and B) Behavioral effects of the cue control and cue conflict rotations. During the cue control trial the behavioral performance of both groups was unaffected, as illustrated by the equivalent short latencies to find the reward [A, latency: *t*(17) = 0.08, *p* = 0.94] and the low number of errors made prior to obtaining reward [B, errors: *t*(15) = 0.07, *p*<0.93]. In contrast, during the cue conflict trial only animals in the visuospatial group were severely disrupted by this manipulation [latency: *t*(17) = 10.6, *p*<0.00001; digs in non-rewarded cups: *t*(17) = 3.87, *p*<0.0007]. Histograms show trial mean±SEM. (C and D) Color-coded rate maps showing firing activity of CA1 pyramidal cells in response to cue rotations. Cartoon placed below each rate map indicates the position of the reward (red circle) and the direction of rotation of the visuospatial environment (90° counterclockwise (CCW), or clockwise (CW)) in each trial. Color map is the same as that shown in Figure 2. (C) Upper panel: Only cells from animals in the visuospatial group displayed concomitant rotations of the fields when the visuospatial cues in the environment were rotated. Middle and bottom panels: Cue rotations had no effect on the firing pattern of unstable cells from animals in the olfactory group. This was the case for cells that exhibited disorganized firing activity as well as those in which firing activity was locked to the reward-associated odor. (D) Rate maps showing place fields of a stable cell recorded in the olfactory group. Cue control and cue conflict experiments [day 4, session 7, T2 and T3 respectively] did not produce re-mapping of the fields in spite of the physical rotation of the environmental cues. On day 5 (session 8), the cue control and cue conflict experiments were replicated on T1 and T2, and on T3 the animal was tested in the dark with the visuospatial cues covered with black paper. As was the case with the first control trials, the fields remained unchanged. Olf, olfactory; T, trial; VS, visuospatial.

In the same manner in which we determined that animals in the olfactory group attended to the reward-associated odor, we next examined whether animals in the visuospatial group attended to the visuospatial cues in the arena to guide their behavior during navigation. To that end, we ran two additional control trials on day 4 (session 7) known as cue control and cue conflict experiments [34]–[37]. These controls were run only once in an order that prevented extinction of the learned association (see Materials and Methods). In the cue control trial, we disrupted the relationship between the distal cues (any fixed cue outside the training environment, e.g., room door) and the reward location, leaving intact the relationship between the proximal visuospatial cues on the wall of the cylinder and the reward location. We rotated the platform with the cups filled with scented bedding and the cylinder with the visual cues in unison 90° counterclockwise. We found that the cue control rotation did not affect behavioral performance in the visuospatial group, supporting the idea that these animals do not attend to cues outside the training environment to find the reward. Similarly, olfactory animals were not affected by this manipulation, since this group learned to attend to a particular odor inside the cylinder rather than any spatial landmark (Figure 6A and 6B) [latency to find the reward (s): visuospatial: 40.22±15.20; olfactory: 37±13.98; errors: visuospatial: 0.14±0.05; olfactory: 0.11±0.04].

In the cue conflict trial, we disrupted the predictive value of the proximal visuospatial cues on the walls of the cylinder and the reward location. To that end, we rotated the platform and cups 90° counterclockwise while rotating the cylinder with the visual cues 90° clockwise. At the behavioral level, we found that animals in the visuospatial condition were severely impaired by this manipulation, whereas animals in the olfactory group were not (Figure 6A and 6B). Visuospatial animals were impaired in finding the reward because, contrary to their expectations, the visuospatial information no longer predicted reward location. This was evident in the latency to find the reward and the number of digs in the nonrewarded cups made before finding the reward, which were dramatically increased in the visuospatial but not the olfactory group [latency to find the reward (s): visuospatial: 787.75±297.94; olfactory: 60.44±22.84; digs in nonrewarded cups: visuospatial: 6.13±2.31; olfactory: 0.22±0.08]. These data provide support for the idea that in the visuospatial group attention to the visuospatial landmarks guides task performance.

At the cellular level, we found that all cue rotations produced concomitant rotations of place fields in animals in the visuospatial group without affecting the firing patterns of cells in the olfactory group (Figure 6C) (correlation comparing place fields during control trials using the physical rotation of the cues as reference frame for analysis: visuospatial: *r* = 0.40±0.03; olfactory: *r* = 0.16±0.05; see Materials and Methods, Rotational Analysis section). In this latter group, the great majority of the neuronal representations shifted in an unpredictable way or re-mapped according to the location of the reward-associated odor (Figure 6C, middle and bottom panel, respectively). Furthermore, only place fields recorded in cells in the visuospatial group displayed angular rotations that approximated the physical rotation of the visuospatial environment (cue control: visuospatial: 90°±8; olfactory: 153°±41, *p*<0.05; cue conflict: visuospatial: 97°±12; olfactory: 137°±49, p<0.05, unpublished data). These results indicate that neuronal responses are locked to the visuospatial landmarks only in those animals that attended to these cues.

### Stable Representations within the Olfactory Group Are Not Driven by Visuospatial Landmarks in the Environment

We found that attending to a spatially shifting odor compromises place field stability in the great majority of cells recorded in the olfactory group (83%, 51 cells). However, a small subset of cells from this group (17%, ten cells, three animals) displayed well-defined, location-specific firing that was stable in the long term (Figure 6D). Further analysis of the characteristics of these stable place fields during the cue control and cue conflict experiments described above as well as additional trials performed in the dark, showed that they were drastically different from the stable fields recorded in the visuospatial condition. Specifically, after all cue rotations the majority of these representations (70%) did not follow the rotation of the visuospatial cues but rather continued firing in the exact same location. In addition, we also tested one animal from which we recorded most of the stable cells (*n* = 6) in the dark with the walls of the training cylinder covered with black paper to prevent the animal from seeing the cues (Figure 6D **,** bottom panel). Under all conditions, cue rotations, and trials in the dark, the fields did not re-map. This is consistent with the hypothesis that, in these cells, neural activity was not driven by the visuospatial cues on the wall of the cylinder or distal cues outside the room. Moreover, since during the trials in the dark location-specific firing was observed before the animals were able to touch the empty cups, and therefore could not use them as object landmarks (unpublished data), the most parsimonious explanation to account for these results is that the firing activity of these neurons was driven by self-movement (idiothetic) information. These results support the idea that when animals learn to ignore the visuospatial cues in the environment, these cues no longer drive neuronal activity in the dorsal hippocampus.

### Visuospatial Task-Performance during Active Exploration Increases Neuronal Synchronization

Thus far, we have examined how task contingencies that engage attention and learning mechanisms to different environmental cues affect the long-term retrieval of information at the single-neuron level. A remaining question is whether the observed changes in retrieval stability are paralleled by changes in the network properties of the cells involved in the encoding of the representations associated with each task. To address this, we looked at neuronal synchronization, a phenomenon by which an assembly of neurons fires together or their firing activity is locked to a particular local oscillation. Neuronal synchronization has been studied primarily in the early stages of sensory processing, where it can lead to an amplification of sensory signals [38]. This suggests that synchronization may mediate attentional effects by biasing information processing in favor of task-relevant information [30]. Furthermore, it has been recently shown that increases in neuronal synchronization correlate with proper memory retrieval [39],[40], suggesting that a mechanism of signal amplification is necessary for proper encoding of information.

Neuronal synchronization can be achieved in two ways: (1) through an increase in the local field potential (LFP) spectral power at a specific frequency, or (2) through increased locking of the spiking activity to specific phases of the local rhythmic oscillatory activity (phase locking). To test if any of these possibilities occurred within the context of our two tasks, we analyzed neuronal synchronization in a subset of animals from which we recorded both the LFP and unit data throughout training (visuospatial: *n* = 3, olfactory: *n* = 5). This analysis was performed during periods of navigation when we could reliably obtain a measure of the LFP, but was not possible during periods of digging and sniffing, because the animals' headstages constantly touched the edges of the cups producing electrical artifacts that limited our ability to accurately measure the LFP.

We first calculated the power in the local field potential at a low frequency (0–20 Hz) that primarily overlapped with the theta band (hereafter called theta) and a medium frequency (20–60 Hz) that overlapped with the gamma band (hereafter called gamma). These two frequencies were selected because they have been implicated in cognitive states, including attention and memory in humans and nonhuman primates [30],[40]–[44]. We found that during navigation, the overall power of theta was higher than the overall power of gamma in the LFP (Figures S5B and S5C; theta: visuospatial = 0.35±0.03, olfactory = 0.33±0.07; gamma: visuospatial: 0.27±0.03, olfactory: 0.22±0.02), consistent with the prominent role of theta during navigation [45]. Importantly, there were no significant changes in the overall power of gamma or theta frequencies across sessions or between the groups [power of gamma: group: *F*(1,25) = 0.12, *p* = 0.74; session: *F*(5,35) = 0.22, *p* = 0.95; interaction: *F*(5,35) = 0.56, *p* = 0.73; power of theta: group: *F*(1,25) = 0.70, *p* = 0.43; session: *F*(5,25) = 0.61, *p* = 0.62; interaction: *F*(5,25) = 1.8, *p* = 0.14] (Figures S5B and S5C). These data indicated that the power of theta and gamma was not affected by group condition or training, remaining constant across sessions in both groups.

**Figure 7:**
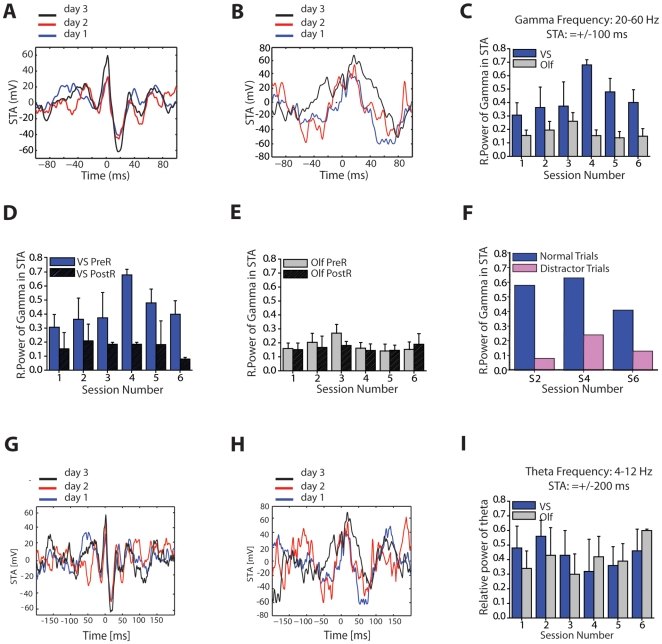
Increase in spike phase locking in animals trained in the visuospatial task. (A and B) Spike-triggered average (STA) generated using a ±100 ms time window on days 1 (blue line), 2 (red line), and 3 (black line) in a visuospatial (A) and olfactory (B) animal. The STA for the animal in the visuospatial group has a clear oscillatory component with a periodicity of about 20 ms (50 Hz). Such a high-frequency oscillatory component is not seen in the STA of the animal in the olfactory group. (C–F) Relative power of gamma (20–60 Hz) in the ±100 ms STA across sessions. (C) In the visuospatial group the relative power of gamma, which reflects phase locking to gamma oscillations, increases during the first 40 s of each trial being maximal after animals reach asymptotic levels of performance. No increase in relative power is observed in the olfactory group. (D) The increase in relative power observed in the visuospatial group during the initial part of the trial (0–40 s) is not present in the last post-reward segment of the trials (860–900 s). (E) In the olfactory group the relative power of gamma does not change before or after obtaining the reward. (F) A distracter (intermittent flashing lights) decreased phase locking in trials 2, 4, and 6 in comparison to normal trials in which no distracting stimuli were presented. (G and H) Same STA examples as those shown in (A and B) generated with a ±200 ms time window. (I) Relative power of theta (4–12 Hz) in the ±200 ms STAs across sessions. In both groups the relative power of theta is relatively high, consistent with the moderately high power of theta in the LFP (Figure S5C), which is characteristic of hippocampal cells during periods of movement. There were no significant differences between the groups or across training trials. LFP, local field potential; Olf, olfactory; PostR, post-reward; PreR, pre-reward; S, session; VS, visuospatial.

Second, we examined if spiking activity was instead preferentially locked to a particular phase of the local oscillatory activity. We computed for each animal the spike-triggered average (STA) of the local field potential. The STA was generated by averaging the activity of the local field potential over time windows of ±100 ms centered on each triggering spike (see Materials and Methods). If spikes were not locked to a particular oscillatory phase in the local field potential, the STA would show no pattern; whereas spike phase locking would produce a synchronized signal in the STA, corresponding to the oscillations generating the locking. Figure 7A and 7B show typical examples of the STA for a visuospatial and an olfactory animal computed during the initial 40 s of the trials. While spikes in the visuospatial animal occurred in synchrony with a high-frequency oscillation (50 Hz), there was no such effect in the animal in the olfactory group.

To quantify if there was a phase-locking effect at different frequencies across animals, we then computed the relative power of the theta and the gamma bands in the STA. No significant differences were observed between the groups or across training trials in the analysis of the relative power of theta [visuospatial: 0.38±0.14; olfactory: 0.33±0.13; group: *F*(1,28) = 0.03, *p* = 0.86; session: *F*(5,28) = 0.40, *p* = 0.85; interaction: *F*(5,28) = 0.43, *p* = 0.82, unpublished data]. This negative result was also found when we used a more restricted frequency range (4–12 Hz) and longer time window for analysis (±200 ms). In both cases, the relative power of theta was moderately high, consistent with the relatively high theta power in the LFP (Figure S5C), but no significant differences between the groups or across training trials were present (Figure 7I) [group: *F*(1,23) = 0.01, *p* = 0.92; session: *F*(5,23) = 0.32, *p* = 0.90; interaction: *F*(5, 23) = 0.45, *p* = 0.81].

We then examined the relative power of the gamma band and found that animals in the visuospatial group showed a gradual relative power increase that was significant after animals learned the task (Figure 7C) [group: *F*(1,26) = 11.13, *p*<0.02; interaction: *F*(5,26) = 2.8, *p*<0.04, groups were significantly different on sessions 4, 5, and 6, *p*<0.05, but not on sessions 1, 2, and 3, *p*>0.05]. The increase in phase locking in the gamma band of the LFP was dependent on the time point during the trial when this effect was measured. In the visuospatial group, this increase was specific to the initial 40-s pre-reward segment of the trials (average latency to find the reward after asymptotic learning: 54 s), remaining low during last 40 s of the trial when the animals had completed the task, and were therefore less likely to attend to the task contingencies and environmental cues (Figure 7D) [group: *F*(1,5) = 506.3, *p*<0.03]. The same comparison did not show any differences in the olfactory group (Figure 7E) [group: *F*(1,9) = 0.06, *p* = 0.83; see also Figure S5D and S5E, showing analysis of longer frequency ranges (20–90 Hz) and time of analysis (0–80 s)]. Even though we observed an increase in the relative power of gamma in the visuospatial group, this increase was not paralleled by an overall increase in the power of the LFP in this frequency. This suggests that the enhancement in relative power in the STA is resulting from increased phase locking in the gamma frequency band. There are two reasons why spikes can lock without causing an increase in their LFP oscillation power: (1) only a small fraction of spikes are actually locking at any given time, and (2) the locked spikes are not very periodic.

To further corroborate that the differences between the groups in the relative power of the STA reflected increased neuronal synchronization, we also analyzed the spike field coherence as described by Fries et al. (2002) [46] (see Materials and Methods). The spike field coherence (SFC) has the advantage of being independent of the fluctuations in the amplitude of the LFP and the spike rate, which makes it a more sensitive measure of synchronization. Using this approach, we found that during periods of navigation when the animals were actively searching for the reward, animals in the visuospatial group displayed higher neuronal coherence than animals in the olfactory group (Figure S5A; average SFC: visuospatial: 0.43±0.03; olfactory: 0.21±0.01; *F*(1,23) = 9.24, *p*<0.03; session number and interaction: not significant). Furthermore, similarly to what we observed in the analysis of the relative power, the increase in SFC observed in the visuospatial group was absent during the last 40 s of the training trial, after animals have found the reward and were no longer attending to the environment [average SFC: visuospatial: 0.16±0.03; olfactory: 0.19±0.02; unpublished data; *F*(1,27) = 0.16, *p* = 0.70, effect of sessions and interaction also not significant *p*>0.05; unpublished data].

Finally, to test whether the observed changes in neuronal synchronization in the visuospatial group were sensitive to manipulations that normally disrupt attention, we examined the effect of a distracter (intermittent flashing lights; one animal, three cells). This manipulation was performed in only one of the trials run on sessions 2 (day 1), 4 (day 2) and 6 (day 3). We performed this analysis using the relative power of the STA, because this measure was the most sensitive to the training effects across days. The distracter was presented for 60 s during the initial part of each of these trials. We found that the relative power within the gamma band for these distracter trials was significantly reduced in comparison to the trials when there was no distracter present (Figure 7F) (*t*(2) = 5.92, *p*<0.02). In sum, here we found that in the hippocampus, phase locking shows sensitivity to task demands and distracters, which are the same parameters that have been shown to affect analogous processes in cortical areas [47]. These findings are consistent with the idea that phase locking to gamma oscillations may underlie a similar process of selective attention in both the cortex and hippocampus.

## Discussion

To determine the behavioral conditions that enhance the stable retrieval of memory representations, we recorded from pyramidal neurons in the CA1 region of the dorsal hippocampus while mice acquired a task that required attention to either a visuospatial location or to a spatially shifting olfactory cue to successfully retrieve a food reward. Using this approach, we found that the stabilization of the place field map does not simply depend on a general form of arousal but requires attention to the visuospatial environment. Attending to a spatially shifting olfactory cue generates unstable place fields and leads to the emergence of neuronal representations in response to the reward-associated odor. These odor representations are more prominent and stable during periods of digging and sniffing. Importantly, during navigation, the enhancement of place field stability in the visuospatial group correlated with an increase in phase locking of action potentials to gamma oscillations. The increase in phase locking parallels the rate of task acquisition, is only present when animals are maximally attentive to the task contingencies, and is disrupted by a distracter, suggesting that this form of neuronal synchronization may underlie an attentional mechanism that facilitates processing of task-relevant information. Together these results indicate that in the hippocampus, attention serves to switch which representations are more consistently retrieved from long-term memory.

By recording from the same hippocampal cells over a number of days, we were able to detect a progressive change in neuronal responses as animals learned to attend to either the visuospatial environment or a particular olfactory cue. During navigation, we observed an increase in the stability of place fields in animals trained in the visuospatial task that was absent in the olfactory group. The lack of place field stability in the olfactory group could simply have resulted from exposing the animals to a spatially shifting goal location. However, since the unstable spatial representations persisted during the probe trials, when animals were tested in the same visuospatial environment but in the absence of odors and reward, these results more likely reflect that animals in the olfactory group stopped attending and assigning significance to the visuospatial landmarks in the environment. Additional support for this idea was provided by the cue rotation findings, showing that none of the representations recorded in the olfactory condition were locked to the visuospatial cues in the environment.

During active exploration, the primary firing mode of hippocampal cells is spatial [31],[48]. However, when visual landmarks are not attended, as it happens during periods of walking in the olfactory group, these spatial representations are highly unstable. Nevertheless, place fields emerge in these situations since animals still have to navigate through space, which drives the activity of hippocampal cells. As a result, the emergence of nonspatial task-relevant information might not be so prominent during these periods of active navigation due to retrieval competition. In agreement with this idea, we found that during periods of navigation, the reward-associated odor representations in the olfactory group were only stable in the short-term. However, when animals were digging and sniffing in a fixed cup location, the reward-associated odor representations were robust and stable. These findings are consistent with previous studies showing that task contingencies modulate the retrieval of hippocampal representations [19],[22],[49]. Here we extend this observation by demonstrating that the task-dependent odor representations are stable over the long term, as indicated by the increases in firing rate in response to the reward-associated odor during stationary periods, and these represenations can be evoked at multiple spatial locations within the same trial. These demonstrations are important because previous physiological studies of the dorsal hippocampus only recorded nonspatial correlates for very brief periods of time [20] or after the animals acquired the task [19],[20], without evaluating the stability of these representations over time. Furthermore, the encoding and retrieval of nonspatial correlates were only tested in one spatial location per trial [19],[20],[50]–[52], confounding the interpretation of whether these representations were encoded within specific spatial coordinates or independently of them.

During periods of digging and sniffing, we did not observe any firing rate changes among the different cups in the visuospatial group. This is not surprising, because during these periods, animals are confined to the cups locations in the close proximity of the odors—the cues that these animals have learned to ignore—and are not processing the visuospatial environment. It is possible that transient responses to the rewarded spatial location occur as animals approach the rewarded cup, as it has been previously demonstrated in other studies where animals display prospective coding of rewarded goal locations [6],[41]. However, these transient responses would be very difficult to evaluate in the open field, because animals approach the cups from many angles and paths. Similarly, since in our study the reward was hidden and its retrieval was not time stamped to the spike data, we could not determine whether cells recorded in animals in both groups displayed transient changes in firing rate to the reward itself. It would be interesting to determine in future studies how transient changes in neuronal activity in response to the rewarded location or the reward itself correlate with the stability of task-relevant representations.

### Attending to Learn and Learning to Attend

We found that the physiological changes associated with the long-term retrieval of both the visuospatial and olfactory representations correlated with the rate of task acquisition. Yet, within the context of our tasks, it is very hard to disentangle the relative contribution of learning versus attention in mediating these effects. The interdependence between learning and attention has been extensively documented in studies showing that what an animal attends to is modulated by what an animal has learned and vice versa [53]–[56]. This is specially the case for selective attention, since this process requires that animals learn to attend to the relevant sensory dimension and ignore the irrelevant one [55],[57]. In this study, this is evident as animals switch from attending to all the cues in the environment, e.g., the cups, odors, and visuospatial cues, to selectively attending to the relevant sensory dimension, here visuospatial or olfactory cues. Thus, only when the dimension-specific selective attention process emerges as a result of learning there is stable retrieval of the encoded trace. Therefore, we posit that it is the selective engagement of both learning and attention to the relevant task-dimensions that leads to proper long-term stable retrieval of hippocampal representations.

This view is consistent with recent imaging findings in humans showing that the retrieval of long-term spatial memories of object locations facilitates spatial orienting and attention to those particular targets [56]. Subjects who had previously learned and memorized the location of objects in complex scenes were much faster in orienting to and locating those objects than subjects with similar experiences with complex scenes but without the memory of the object location. Furthermore, this form of learning-guided attention involved the activation of the hippocampus during the orienting phase, demonstrating the interdependence of attention and learning processes in this region.

The interdependence between learning and attention mechanisms has also been found in cortical areas that have been traditionally associated with attention processes. For example, cortical neuronal synchronization, a proposed mechanism of selective attention required for the encoding of task-relevant information [29], is enhanced in response to attended stimuli and diminished by unattended ones or distracters [30],[47],[58]. In addition, cortical synchronization also displays sensitivity to task performance, which is a correlate of learning [59]. Consistent with these data, in our study hippocampal phase locking was decreased under conditions of reduced or disrupted attention, at the end of trials and in the presence of a distracter. Moreover, the enhancement in hippocampal phase locking observed in the visuospatial group paralleled the rate of task acquisition, indicating that hippocampal synchronization is also sensitive to task performance. These similarities suggest that the enhancement in phase locking to gamma oscillations observed in this study might underlie a hippocampus-dependant attentional mechanism that serves to process task-relevant information as it happens in cortical areas.

### Neuronal Synchronization and Place Field Stability

How could neuronal synchronization in the gamma frequency lead to stabilization of place fields? Like memory, Hebbian forms of LTP require NMDA receptor activity, protein kinase A (PKA), and synthesis of new proteins for proper induction and consolidation [60]–[62]. Since the stability of the place field map also requires the same biochemical cascades [10]–[12], it is thought that this phenomenon represents a correlate of spatial memory that is achieved, at the cellular level, by an LTP-like phenomenon [11]. Such a mechanism could be induced by increases in neuronal synchronization, which have been previously shown to modulate different forms of plasticity [63],[64]. For example, action potentials that correlate with the peak of gamma or theta oscillations lead to LTP, and those that correlate with the troughs lead to long-term depression [65]–[67]. Furthermore, pre and postsynaptic activity occurring within time windows ranging between 10 to 30 ms are optimal for the induction of plastic changes [68],[69]. The same time intervals characterize the cycling patterns of gamma oscillations, suggesting that these oscillations are in a unique position to modulate the effectiveness of action potentials through plastic mechanisms [63]. Since in our study, the increase in place field stability observed in the visuospatial group correlates with an increase in phase locking to the gamma band, we suggest that neuronal synchronization might be a mechanism that serves to increase the signal-to-noise ratio of the relevant visuospatial information through an enhancement of synaptic connections. It still remains to be determined what physiological changes follow the initial synchronization in order to produce long-lasting alterations at the synaptic level, how different frequencies might contribute to enhance task-relevant information at different time points during acquisition, and how different behavioral paradigms and/or states may affect this phenomena. Unfortunately, within the context of our paradigm, we could not obtain a reliable measure of synchronization during periods of digging and sniffing, but in future studies, it would be interesting to determine whether neuronal responses to nonspatial cues could also affect synchronization in the dorsal hippocampus.

In summary, by recording from the same neurons over a period of several days, we found that learning to attend to the visuospatial environment enhances both the stable retrieval of spatial representations and neuronal synchronization, whereas learning to attend to a shifting olfactory cue increases the retrieval of reward-associated odor representations. These results are consistent with the idea that the interaction between learning and attention strongly influences long-term memory in the dorsal hippocampus.

## Materials and Methods

### Subjects

Male C57/BL6 mice (10–16 wk old) were food deprived after recovery from surgery to 85% of their free body weight prior the start of the behavioral experiments (2 wk after surgery). The mice were tested during the light phase of a 12-h light/dark cycle. The methods described have been designed to minimize animal number and discomfort and were conducted according to the National Institutes of Health standards using protocols approved by Columbia University IACUC.

### Surgical Procedure

Mice were anesthetized with a mixture of ketamine (100 mg/kg) and xylazine (7 mg/kg) administered i.p. (0.1 ml/kg) and placed in a flat skull position in a stereotaxic frame (David Kopf Instruments). Animals were implanted with a drivable four-tetrode headstage (each wire 25-µm nichrome, California Fine Wire). Recording electrodes were placed just above the dorsal hippocampus. Coordinates for implantation from bregma in mm: AP, −1.8; ML, 1.8; from dura: DV, −0.9.

### Apparatus

The training arena consisted of a white wood cylinder 50 cm in diameter and 50 cm in height. The cylinder was placed on a fitted white wood platform. The apparatus was visually isolated from the rest of the laboratory by a concentrically placed black curtain (200 cm in diameter and 220 cm in height).

### Behavioral Training

After recovery from surgery, animals were shaped to dig for food in their home cage. This shaping was performed by feeding the animals once daily with a 3-g food pellet buried under sterilized unscented woodchips in a medicine cup (Henry Schein) placed in their home cage. This procedure encouraged mice to dig in the cup in order to obtain the food.

Behavioral training started only once stable single unit recordings were achieved (see below). Scented woodchips were introduced on the first day of training [scented bedding: 5 g of powder spices (cinnamon, cumin, ginger, or cloves; The Great American Spice Company) in 500 g of woodchips (animals' normal home cage bedding)]. Animals were taught the visuospatial or olfactory task-contingencies by priming them with respect to the location of the reward on trials 1 and 2 during the first session. This was achieved by placing the reward on top of the cup (three Cocoa Rice Krispies cut in half, Kelloggs). After this, the reward was always buried 2 cm below the surface in disperse locations. Having the reward dispersed inside the cup in little pieces avoided extinction of the reward-searching and digging behaviors during the 15-min training trials. At the end of each trial, the animals were removed from the test chamber and placed inside a black beaker, while still being tethered to the recording equipment. This beaker was positioned 20 cm away from the test chamber. During this intertrial period (ITI = 2 min), a clean platform with new clean cups filled with scented bedding was placed in the training arena. At the end of the experiment, all platforms were cleaned with ethanol to remove odor trails. Mice were always introduced into the training arena in the same orientation facing the same visual cue. Animals received two training sessions per day (ISI = 7–8 h (daytime) or 12–14 h (overnight).

On the final day of recording (day 4, session 7), a series of control experiments were performed. During cue rotation trials, animals were introduced in the arena facing the same cue that they had faced prior the rotation. Thus, if the cylinder was rotated 90° counterclockwise, the animal was rotated the same angle when it was introduced in the environment. Each control experiment (cue control, cue conflict, and same odor experiments) produced different level of disruption to normal task performance, depending on the condition in which animals were trained. This happened because the control trials affected the task-contingency rules. To avoid this, we varied the order of presentations. The sequence of control presentations for animals in the visuospatial group was: T1: same odor, T2: cue control, T3: cue conflict. For animals in the olfactory group the sequence was: T1: cue control, T2: cue conflict, T3: same odor. The same odor control was run in a subset of animals in the visuospatial and olfactory tasks, because this experiment was added after we observed the emergence of cells that shifted with the position of the reward-associated odor in the olfactory group (visuospatial, *n* = 5; olfactory: *n* = 7).

To corroborate the lack of effect of the cue rotation experiments on “idiothetic” cells, we repeated the controls in one animal (i71) on day 5. In this animal, additional training trials were conducted in between the control sessions to avoid extinction of the original association.

### Behavioral Analysis

We recorded head position using two tracking systems: (1) The Discovery tracking system (Datawave Technologies), which tracks light-emitting diodes (LEDs) positioned on the head stage of the animal. This provided an accurate measure of the position and speed of the animal at any time during each trial. Importantly, this tracking system was linked to our spike acquisition software and hence allowed us to differentially analyze spike activity during periods of movement and immobility at any location in the environment. (2) The LimeLight video tracking system (ActiMetrics) was used for detailed analysis of behavior off-line. LimeLight permitted user-defined behaviors to be scored while viewing the trial from a stored digital video image. The off-line scoring of the behavior recorded in LimeLight as well as in video tapes was done blind, with the observer unaware of the objectives of the experiment. The scored behaviors included: latency to find the reward, digging time in each of the cups, and errors before finding the reward in the designated cup.

### Physiology: Long-Term, Single-Unit Recording of CA1 Pyramidal Neurons in Dorsal Hippocampus

We obtained behavioral and physiological data from 23 C57Bl6 male mice. All cells displaying stable recordings were included in the analysis regardless of whether or not they had a well-defined spatial field, thus avoiding artificial selection bias for analysis. At the beginning of training (day 1) we properly isolated 58 cells (12 animals) in the visuospatial group, and 64 (11 animals) cells in the olfactory group. At the end of the experiment (day 4), we recorded 24 cells (six animals) in the visuospatial condition and 41 cells (eight animals) in the olfactory condition from the original pool of cells. In cases where we lost cells after 1 or 2 days (five animals in the visuospatial condition and three animals in the olfactory condition), we used the partial data for analysis of firing fields during early acquisition.

In our recording setup the microdrive cemented on the animal's skull was connected to tethered head stage with a unit gain amplifier for each wire and a red LED for tracking the position of the animal's head. The microdrive and the LED were connected to a long cable plugged to a commutator, which allowed mice to move freely in the arena. The fixed side of the commutator was connected to a distribution panel. Units were amplified about 10,000 times using an eight-channel amplifier (Neuralynx) and band-pass filtered at 300–10,000 Hz. The amplifier output was digitized at 20–40 kHz. The position of the animal and electrophysiological data were recorded by a Datawave Workstation (Datawave Technologies), which recorded 1 ms of firing activity at 20–40 kHz each time the voltage signal exceeded an experimenter-defined threshold. Before the beginning of all experiments units were isolated on-line (DataWave Discovery) to facilitate visualization of the cells during the experiments and provide a quick way to assess recording stability. At the end of each session, all units were re-cut offline to ensure that the quality of the recordings had not changed significantly from preceding sessions.

### Unit Discrimination

Beginning 2 wk after surgery, neural activity from each wire was screened daily. If no hippocampal pyramidal cells were identified the electrode bundle was advanced by 20-µm steps daily. We found that lowering the electrodes in small steps increases the stability of the recordings [11]. Every animal was screened several times before recordings (range 9–35) prior to the start of the experiment.

Pyramidal CA1 units were identified by their phenotypic firing pattern characterized by a tendency to fire in “complex spikes,” bursts of 2–7 spikes of decreasing extracellular amplitude that fire at short (5–7 ms) inter-spike intervals. With a noise level about 40 µV, we only accepted units for analysis with signals above 200 µV and spike width of about 300 µm.

After the experiment was complete (day 4), all the recorded sessions were analyzed blind. Animal names and file numbers were changed (but their sequence was kept the same) and the blind broken only after the analysis was complete. Unit quality was analyzed offline using Autocut (Datawave Technologies). We only accepted cells for analysis if they formed isolated clusters that had clear Gaussian ellipses exhibiting minimal overlap between neighboring clusters or noise (Figure 4C and 4D). All clusters were inspected to ensure that the complex spike interval (4–7 ms) was the largest bin in the autocorrelogram, and that none of the clusters exhibited events within the 2-ms spike refractory period. Criteria for long-term stable recording were: (1) The unit must have the same cluster boundaries in two long-term sessions (ISI = 7–8 h or 12–14 h, and (2) the waveforms obtained on all four wires of the tetrode must be identical in all sessions recorded (Figures 2 and 3). Experiments started when both these criteria were met.

### Data Analysis

First the recording stability and quality of the cells was determined in each of the four trials in sessions 1 to 7 for every animal run in the study (see above). Unit activity was recorded during all the trials (T0–T3), but not during the inter-trial intervals (2 min) and analyzed using previously published methods [10],[27],[36]. Briefly, the area of the training environment was subdivided into two 30×30 pixilated grids (each pixel = 2.2×2.2 cm). Using these grids, we generated two arrays of data, one containing the total number of spikes in each pixel (*spike map*) and the other the total time the mouse spends in each pixel. Dividing the spike array by the time array generated spike rate maps, which are two-dimensional representations of the training environment with each pixel color-coded for time-averaged firing rate. Yellow = no firing activity, white = unvisited regions. Increased color hue represents higher firing frequency. The generation of the rate maps and all the quantification analysis described below were done using software developed by Matt Stead and Naveen Agnihotri based on the analysis package used by Robert Muller (SUNY, Brooklyn). Place field stability was measured by performing pixel by pixel Pearson R cross-correlations. In cases where all the cells from an animal were lost before the completion of the experiment, we adjusted the standard errors to reflect the number of remaining animals in all subsequent sessions.

We used four measures for quantitative analysis of the properties of the firing fields: *coherence*, *information content*, *firing frequency*, and *size* [22],[27]. Coherence was measured by calculating the Z transform of the correlation between the firing rates in each pixel and the average firing rates of the eight nearest-neighbor pixels. Information content was calculated by subdividing the training arena into a 30×30 pixilated grid in the same manner as described for the generation of spike and rate maps. The amount of information that each bin generated by the grid conveys about the location of the animal will be calculated using the formula: 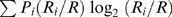, where *P_i_* is the probability of occupancy in bin *i*, *R_i_* is the average firing rate for bin *i*, and *R* is the overall mean firing rate. Field size is reported in pixel units and is calculated from areas containing at least four contiguous pixels where the cell fired. The total firing frequency was calculated by dividing the total number of spikes by the time the animal was moving (speed 2 cm/s) during the session.

#### Stability threshold measure

To determine whether a cell was stable, or not we used the cutoff correlation value 0.2. This value was selected because before training both the visuospatial and olfactory groups displayed baseline stability values close but below 0.2. After training, cells displaying stability values below 0.2 were considered unstable, whereas cells displayed stability values above 0.2 were considered stable. This method provided an unbiased way to classify cells before and after training.

#### Classification of olfactory representations during navigation

Once cells were classified as stable or unstable using the spatial reference frame (see above), we further analyzed the unstable group to determine the percentage of cells that showed unorganized firing versus those that displayed reward-associated odor coding. To this end, we mapped the position of the cups by creating a file that was generated by moving the LEDs over the four cup locations when the animals were not connected. This file was superimposed over the computer generated rate maps and the position of the reward area was marked for each trial. Then, the numbers of fields inside and outside the cup locations were counted in the rate map. A field was defined as a region in which the firing rates of all the pixels were greater than zero, and whose total area equaled or exceeded nine contiguous pixels. Two pixels were considered contiguous only when they shared one side.

#### Rotational analysis performed during navigation

To assess the stability of the reward-associated odor representations during periods of active exploration, we compared successive trials by rotating the raw tracker data and re-computing the spatial analyses with the rotated tracker coordinates. The range of possible rotations was divided equally into 360 1-degree increments. Correlation of spatial information was generated by performing pixel by pixel Pearson correlations of corresponding smoothed rate maps. Smoothing of the rate maps was performed to facilitate identification of a discrete peak in the rotation by the correlation function, which was taken to be the best angular fit. Each pixel in the smoothed maps corresponded to half the rate of the directly corresponding pixel plus half the mean rate of the surrounding 8 pixels. To test the stability of olfactory representations during periods of exploration, we selected the angle that corresponded to the position of the reward, which shifted pseudo-randomly among the four cup locations at 0°, 90°, 180°, and 270° as reference frame for analysis. For the cue control and cue conflict rotations, we used the angle that indicated the physical rotation of the environment (90° clockwise or counterclockwise).

### Analysis of Firing Frequency during Periods of Sniffing and Digging

This analysis was performed by creating a map of each cup that restricted the analysis of firing frequency to that cup location. Only cells that fired during the whole trial at a rate of above 0.05 spikes/s were used in the average. All data were filtered for periods when the animals were moving at a speed of less than 2 cm/s. This low threshold was set to capture head movements during digging (cup diameter: 5 cm). After the data were generated for each animal, we calculated the firing rate ratio of rewarded to non-rewarded cups for each trial. Then, we averaged these ratios across sessions. Ratios above 1 indicated that the cells fired more strongly in response to the rewarded cup than to the non-rewarded cups. Ratios below one indicated the opposite pattern. A similar analysis was conducted to calculate firing rate responses to non-rewarded odors.

### Spike Synchronization Analysis

This analysis was performed in a subset of animals from which we successfully recorded both unit and LFP activity throughout training (visuospatial: *n* = 3, olfactory: *n* = 5). The LFP signal was recorded using one of the tetrode wires from which we obtained unit activity. This signal was referenced to an electrode positioned below the skull in the occipital lobe on the contra-lateral hemisphere to the recording electrode. The LFP data were recorded using a digital filter ranging between 0.1–400 Hz. For analysis, these data were first pre-processed to exclude epochs when the recorded values crossed established thresholds, an artifact introduced by the mouse's head hitting the wall of the environment. Line-noise at 60 Hz was removed from the LFP signal using the Multi-Taper approach outlined by [70]. The Multi-Taper method of spectral analysis allows spectral estimation and signal reconstruction of a time series, which is assumed to have a spectrum containing both continuous and singular components [70],[71]. The tapers are discrete sets of eigenfunctions that solve the variability problem by minimizing leakage at a specific frequency band. The statistical confidence interval was calculated using the (*F*) test. Once statistically significant peaks were isolated in the spectrum, the Multi-Taper approach was also used to reshape the spectrum within a frequency if the *F*-test was found to be significant at that frequency. After filtering the 60 Hz noise using the Multi-Taper method, the signal was band-pass filtered between 1 to 150 Hz, to obtain the LFP. The STA was computed by isolating LFP segments of ±100 ms centered on each triggered spike that were subsequently averaged. For the analysis of theta we also generated STAs using LFP segments of ±200 ms.

The spectrum of the spike-triggered average was calculated using the Multi-Taper method to compute standard Fourier transforms. The relative power of theta or gamma oscillations was computed by calculating the area under the spectrum at that frequency divided by the total area under the spectrum. This normalization allows making comparisons across different days of training. All the relative power analyses were performed on three visuospatial and five olfactory animals. One exception was the relative power of theta using a 200-ms time window in the STA where one olfactory animal did not have sufficient spikes for the analysis (spike filter = 50 spikes) and therefore the olfactory *n* was 4. As a control for the relative power we also computed the power of the theta and gamma oscillations in the LFP.

The spike-field coherence (SFC) was calculated as described by [46]. We computed the power of the LFP segments used in the computation of the STA. Then, these power spectra were averaged to obtain the spike-triggered power (STP). The SFC is the ratio of the power spectrum of the spike-triggered average to the STP. The SFC ranges from 0, which indicates an absence of synchronization, to 1, which indicates complete synchronization. The SFC was computed only for sessions where more than 50 spikes were recorded.

### Verification of Electrode Placement

At the completion of the experiment the animals were deeply anesthetized with a mixture (0.4 ml) of xylazine (100 mg/kg) and ketamine (7 mg/kg). Final electrode positions were marked by passing a 150 µA current for 10 s at positive and negative polarities using a Grass stimulator (Grass Technologies) through the tetrode/s that yielded unit data. The animals were then perfused transcardiacally with 0.1 M PBS followed by 4% (wt/vol) paraformaldehyde made in 0.1 M PBS. The brains were then placed in 4% paraformaldehyde containing 3% ferrocyanide for Prussian blue staining (24 h), and then incubated over night in a 30% (wt/vol) sucrose solution made in 0.1 M PBS for cryoprotection. All brains were cryosectioned (40 µm, coronal) and stained with cressyl violet using standard histological procedures and then cover-slipped with Permount mounting medium (Fisher Scientific).

### Statistical Analysis

Statistical analysis was done as previously described [10],[27]. Briefly, we used parametric statistics including two-way repeated measure ANOVAS where time and group (olfactory versus visuospatial) were the independent variables and the various parameters we measured the dependent variable, *t*-tests or ANOVAS for independent groups when we compared two or more independent groups respectively and paired *t*-tests when we compared two groups and one repeated measure variable. For post hoc analysis we used the Student Newman Keuls method, which is appropriate for multiple comparisons. All statistical analysis was done using Sigma Stat (Systat Software). In all tests, we used the animal as the unit for analysis.

## Supporting Information

Figure S1 **Experimental design and digging time in rewarded and non-rewarded cups. (**A) Experimental design. On day 0, recording stability and quality of cells were determined during free exploration of the arena (see Materials and Methods). Behavioral training commenced the following day. Animals were trained for three consecutive days with two sessions per day (intersession interval = 7–8 to 12–14 h with four 15-min trials per session (ITI = 2 min). The first trial on every session (T0) was a probe trial during which animals explored the test arena in the absence of task contingencies. T0 was followed by three training trials (T1–3). On day 4, session 7, a series of control experiments were performed. (B) Photograph of the training environment during the probe trial (T0). (C and D). Both the visuospatial (C) and olfactory (D) groups showed a gradual increase in digging time in the rewarded cup that occurred with a concomitant reduction in digging time in the incorrect cups. There was no significant difference between animals in the visuospatial and olfactory groups in digging time in the rewarded [*F*(1,10) = 0.03, *p* = 0.87] or nonrewarded cups [*F*(1,10) = 2.58, *p* = 0.14]. Histograms show mean±standard error of the mean (SEM).
(1.87 MB TIF)

Figure S2 **Trail maps.** Representative trail maps recorded during task performance (days 1 to 3) from an animal in the visuospatial group (A) and from an animal in the olfactory group (B). Neuronal activity from cells recorded in both these animals are shown in Figure 2A and 3B, respectively. Note the extensive sampling of the environment in both task conditions. T0 = probe trial, T1 to T3 = training trials.
(6.47 MB TIF)

Figure S3 **Attention to the visuospatial environment affects the spatial properties of place fields.** (A) Long-term center-of-mass shift (COM shift). The COM is a dynamic property of place fields that has been shown to change with experience [72]. We calculated the COM for each cell by determining the *x* and *y* coordinates of the point of highest firing frequency in the place field. COM shifts were significantly different in both conditions [*F*(1,57) = 5.89, *p*<0.03]. However, the decrease in mean values observed in the visuospatial group only showed a trend (*p* = 0.15). (B and C) Percent change from baseline in place field size and firing rate. (B) Field size was not significantly different between the groups [*F*(1,80) = 0.97, *p* = 0.34] but there was a modest decrease in both groups across sessions [*F*(5,80) = 2.66, *p*<0.03]. This happened without an interaction between group and session number [*F*(5,80) = 0.40, *p* = 0.85]. The lack of significant differences between the groups in field size, despite the fact that some fields in the olfactory group became completely disorganized at the end of training, reflected the variability in representational phenotypes observed in this group during periods of navigation. (C) There were no significant differences between the groups or across session in average firing rate [group: *F*(1,80) = 0.22, *p* = 0.64; session: *F*(5,80) = 0.55, *p* = 0.73; interaction: *F*(5,80) = 0.37, *p* = 0.86]. Histograms show mean±SEM.
(0.81 MB TIF)

Figure S4 **During navigation neurons in the olfactory task retrieve different types of representations.** Rate maps of sessions 1–6 showing spatial and olfactory representations recorded from an animal trained in the olfactory group. During some trials the representations recorded from this cell were spatial (session 1 and 3), whereas in others they were locked to the location of the reward-associated odor (session 4). Color map indicates neuronal level of activity. Yellow pixels are regions the animal visited but the cell never fired. Orange, red, green, blue, and purple pixels encode progressively higher firing rates that are auto-scaled relative to the peak firing frequency (shown above each rate map). T0 = probe trial, T1 to T3 = training trials.
(1.48 MB DOC)

Figure S5 **Neuronal synchronicity increases in the visuospatial group without changes in the power of the LFP.** (A). Spike field coherence. This form of synchronicity was enhanced in the visuospatial group in comparison to the olfactory group [*F*(1,23) = 9.24, *p*<0.03]. However, the effect of session and interaction were not significant [session: *F*(5,23) = 1.07, *p* = 0.40; interaction: *F*(5,23) = 0.35, *p* = 0.88]. (B and C) The power of gamma (B) and theta (C) in the LFP showed no clear peak at any point during training and no significant differences between the groups. (D) Extending the gamma frequency band (20–90 Hz) showed the same trend observed in the low gamma frequency band (20–60 Hz). However, the differences between the groups were not significant [main effect of groups: *F*(1,27) = 2.06, *p* = 0.2; sessions: *F*(5,27) = 0.90, *p* = 0.49; interaction: *F*(5,27) = 0.17, *p* = 0.97]. (E) Extending the time of analysis to 80 s also showed the same trend observed during the first 40 s, but the differences between the groups were not statistically significant [main effect of groups: *F*(1,27) = 2.22, *p* = 0.18; sessions: *F*(5, 27) = 0.39, *p* = 0.85; interaction: *F*(5, 27) = 0.63, *p* = 0.68]. Olf, olfactory; VS, visuospatial.
(1.01 MB DOC)

Table S1 **Firing rate across sessions (S1 to S6) over the rewarded (R) and nonrewarded (non-R) odors during periods of digging and sniffing (speed threshold below 2 cm/s).**
(0.55 MB TIF)

Table S2 **Average latency to find the reward across days for the olfactory group and animal B11.** The data show that animal B11 latencies were longer than the average on days 1 and 2, which might have contributed to the fact that this was the only animal that displayed significant firing rate responses to a non-rewarded odor in addition to the reward-associated odor.
(0.24 MB TIF)

## Abbreviations

ISI: intersession interval
ITI: intertrial interval
LED: light-emitting diodes, LFP, local field potential
LTP: long-term potentiation
SEM: standard error of the mean
SFC: spike field coherence
STA: spike-triggered average
