# Correction: Attention Enhances the Retrieval and Stability of Visuospatial and Olfactory Representations in the Dorsal Hippocampus

**DOI:** 10.1371/annotation/d0e5ef6f-d08b-474a-8fde-aeebeee7369d

**Published:** 2010-10-01

**Authors:** Isabel A. Muzzio, Liat Levita, Jayant Kulkarni, Joseph Monaco, Clifford Kentros, Matthew Stead, Larry F. Abbott, Eric R. Kandel

Please view the correct Figure S5 here: 
