# Correction: Attention Enhances the Retrieval and Stability of Visuospatial and Olfactory Representations in the Dorsal Hippocampus

**DOI:** 10.1371/annotation/5e28240c-6186-43eb-b319-79c391d9468a

**Published:** 2010-10-01

**Authors:** Isabel A. Muzzio, Liat Levita, Jayant Kulkarni, Joseph Monaco, Clifford Kentros, Matthew Stead, Larry F. Abbott, Eric R. Kandel

Please view the correct Figure S4 here:
