# Supplementary figures and images for: Attention Enhances the Retrieval and Stability of Visuospatial and Olfactory Representations in the Dorsal Hippocampus
Source: PLoS Biol. 2009 Jun 30;7(6):e1000140. doi: 10.1371/journal.pbio.1000140 (PMC2696347; doi:10.1371/journal.pbio.1000140)

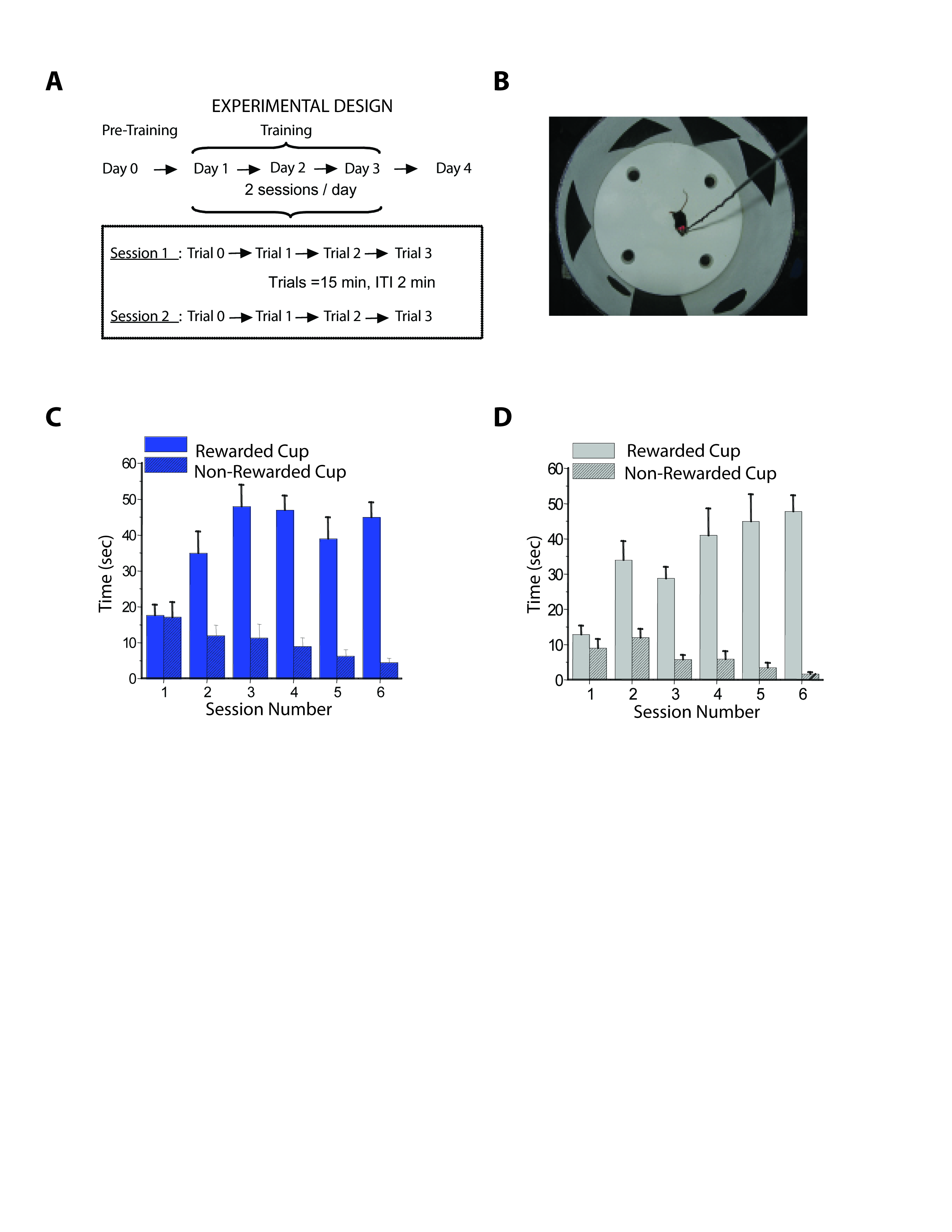

Supplement: Figure S1 — Experimental design and digging time in rewarded and non-rewarded cups. (A) Experimental design. On day 0, recording stability and quality of cells were determined during free exploration of the arena (see Materials and Methods). Behavioral training commenced the following day. Animals were trained for three consecutive days with two sessions per day (intersession interval = 7–8 to 12–14 h with four 15-min trials per session (ITI = 2 min). The first trial on every session (T0) was a probe trial during which animals explored the test arena in the absence of task contingencies. T0 was followed by three training trials (T1–3). On day 4, session 7, a series of control experiments were performed. (B) Photograph of the training environment during the probe trial (T0). (C and D). Both the visuospatial (C) and olfactory (D) groups showed a gradual increase in digging time in the rewarded cup that occurred with a concomitant reduction in digging time in the incorrect cups. There was no significant difference between animals in the visuospatial and olfactory groups in digging time in the rewarded [F(1,10) = 0.03, p = 0.87] or nonrewarded cups [F(1,10) = 2.58, p = 0.14]. Histograms show mean±standard error of the mean (SEM). (1.87 MB TIF) [file pbio.1000140.s001.tif]

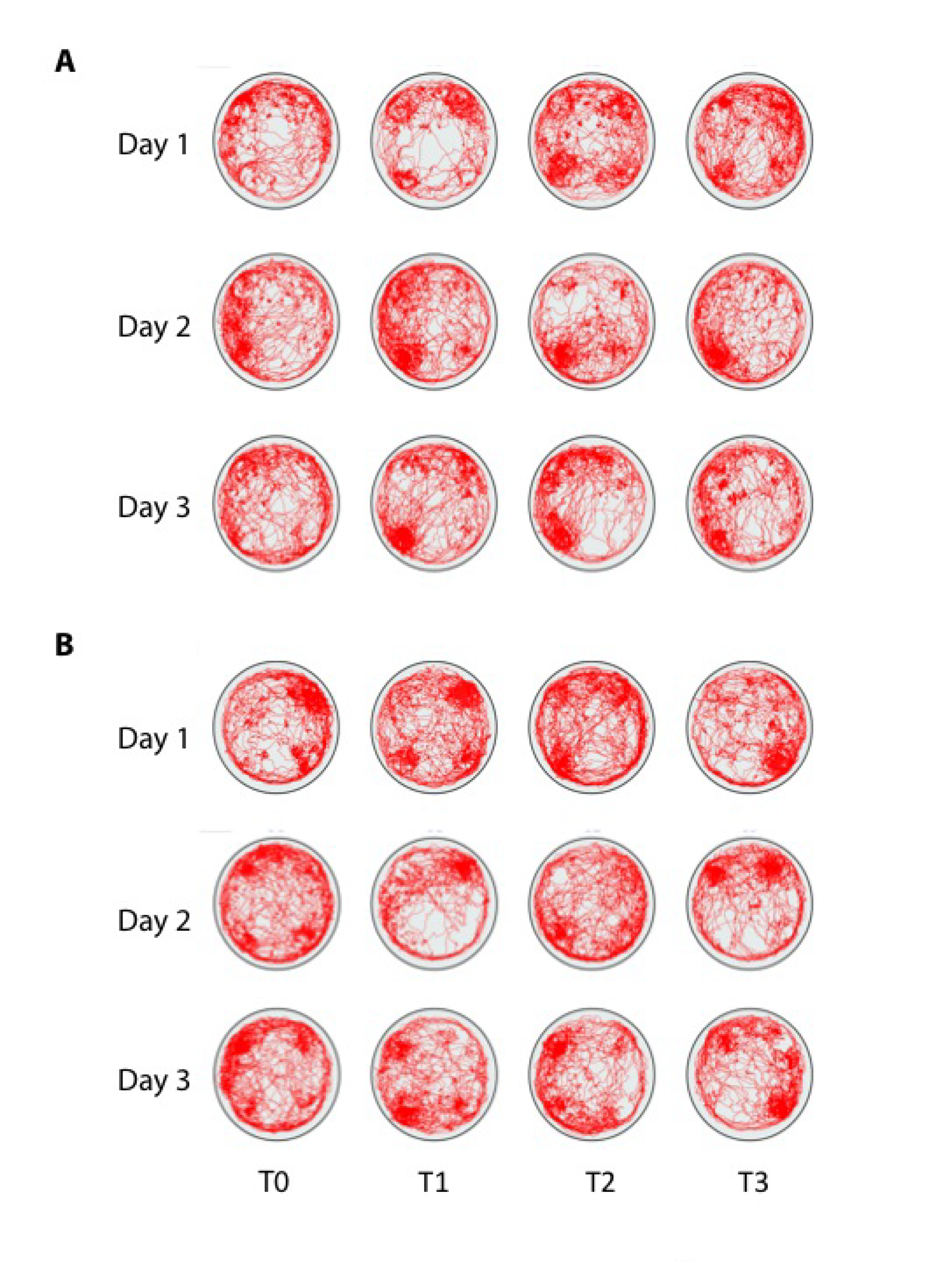

Supplement: Figure S2 — Trail maps. Representative trail maps recorded during task performance (days 1 to 3) from an animal in the visuospatial group (A) and from an animal in the olfactory group (B). Neuronal activity from cells recorded in both these animals are shown in Figure 2A and 3B, respectively. Note the extensive sampling of the environment in both task conditions. T0 = probe trial, T1 to T3 = training trials. (6.47 MB TIF) [file pbio.1000140.s002.tif]

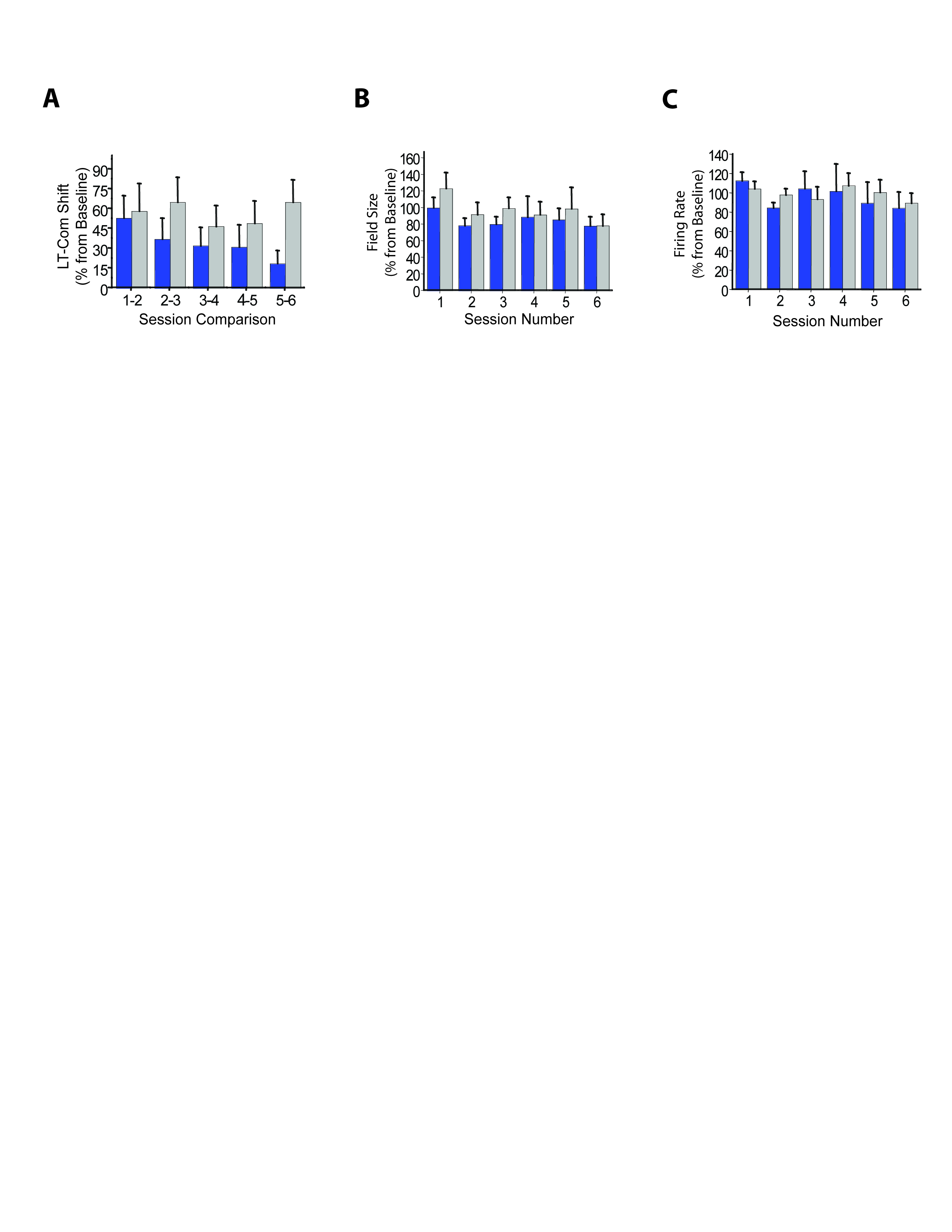

Supplement: Figure S3 — Attention to the visuospatial environment affects the spatial properties of place fields. (A) Long-term center-of-mass shift (COM shift). The COM is a dynamic property of place fields that has been shown to change with experience [72]. We calculated the COM for each cell by determining the x and y coordinates of the point of highest firing frequency in the place field. COM shifts were significantly different in both conditions [F(1,57) = 5.89, p<0.03]. However, the decrease in mean values observed in the visuospatial group only showed a trend (p = 0.15). (B and C) Percent change from baseline in place field size and firing rate. (B) Field size was not significantly different between the groups [F(1,80) = 0.97, p = 0.34] but there was a modest decrease in both groups across sessions [F(5,80) = 2.66, p<0.03]. This happened without an interaction between group and session number [F(5,80) = 0.40, p = 0.85]. The lack of significant differences between the groups in field size, despite the fact that some fields in the olfactory group became completely disorganized at the end of training, reflected the variability in representational phenotypes observed in this group during periods of navigation. (C) There were no significant differences between the groups or across session in average firing rate [group: F(1,80) = 0.22, p = 0.64; session: F(5,80) = 0.55, p = 0.73; interaction: F(5,80) = 0.37, p = 0.86]. Histograms show mean±SEM. (0.81 MB TIF) [file pbio.1000140.s003.tif]

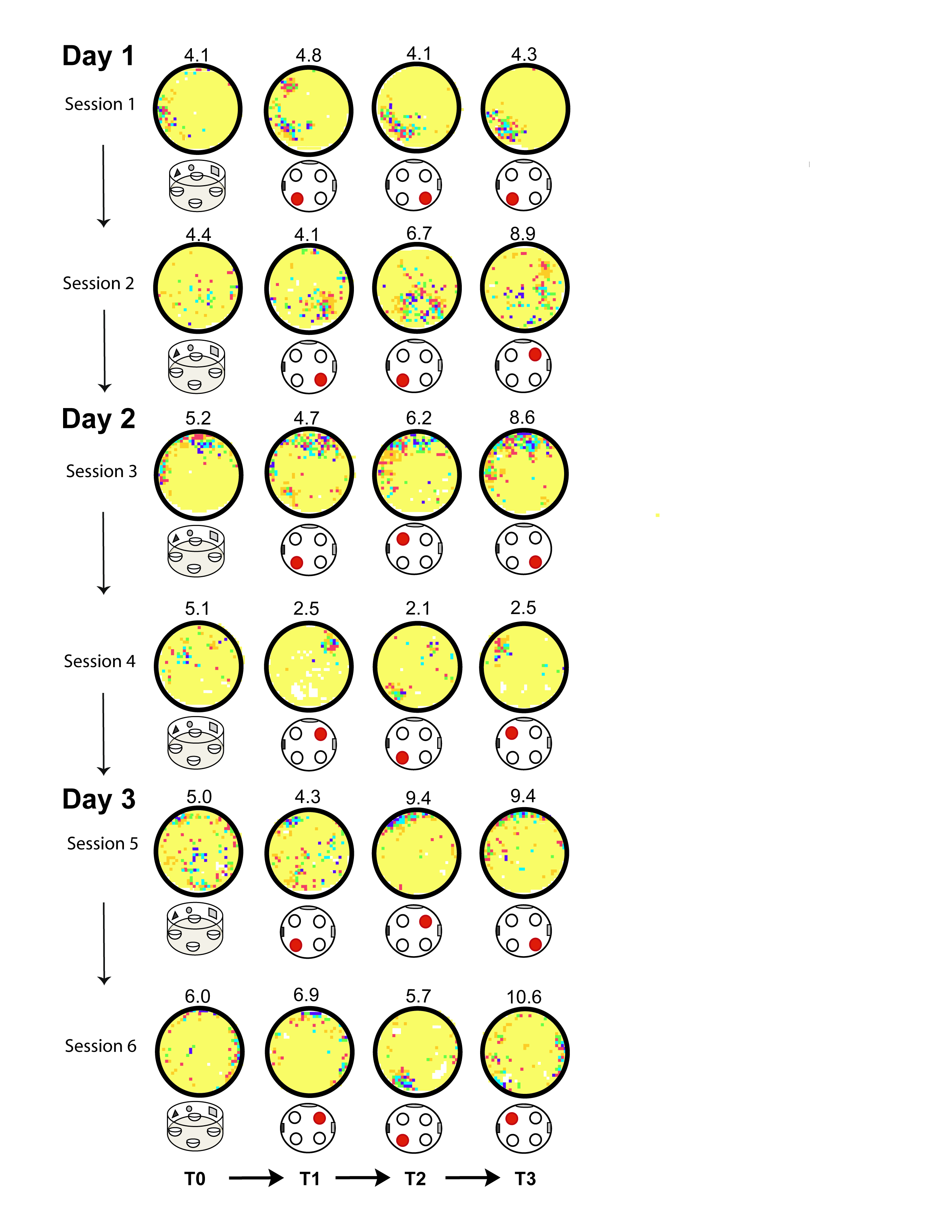

Supplement: Figure S4 — During navigation neurons in the olfactory task retrieve different types of representations. Rate maps of sessions 1–6 showing spatial and olfactory representations recorded from an animal trained in the olfactory group. During some trials the representations recorded from this cell were spatial (session 1 and 3), whereas in others they were locked to the location of the reward-associated odor (session 4). Color map indicates neuronal level of activity. Yellow pixels are regions the animal visited but the cell never fired. Orange, red, green, blue, and purple pixels encode progressively higher firing rates that are auto-scaled relative to the peak firing frequency (shown above each rate map). T0 = probe trial, T1 to T3 = training trials. (1.48 MB DOC) [file pbio.1000140.s004.doc]

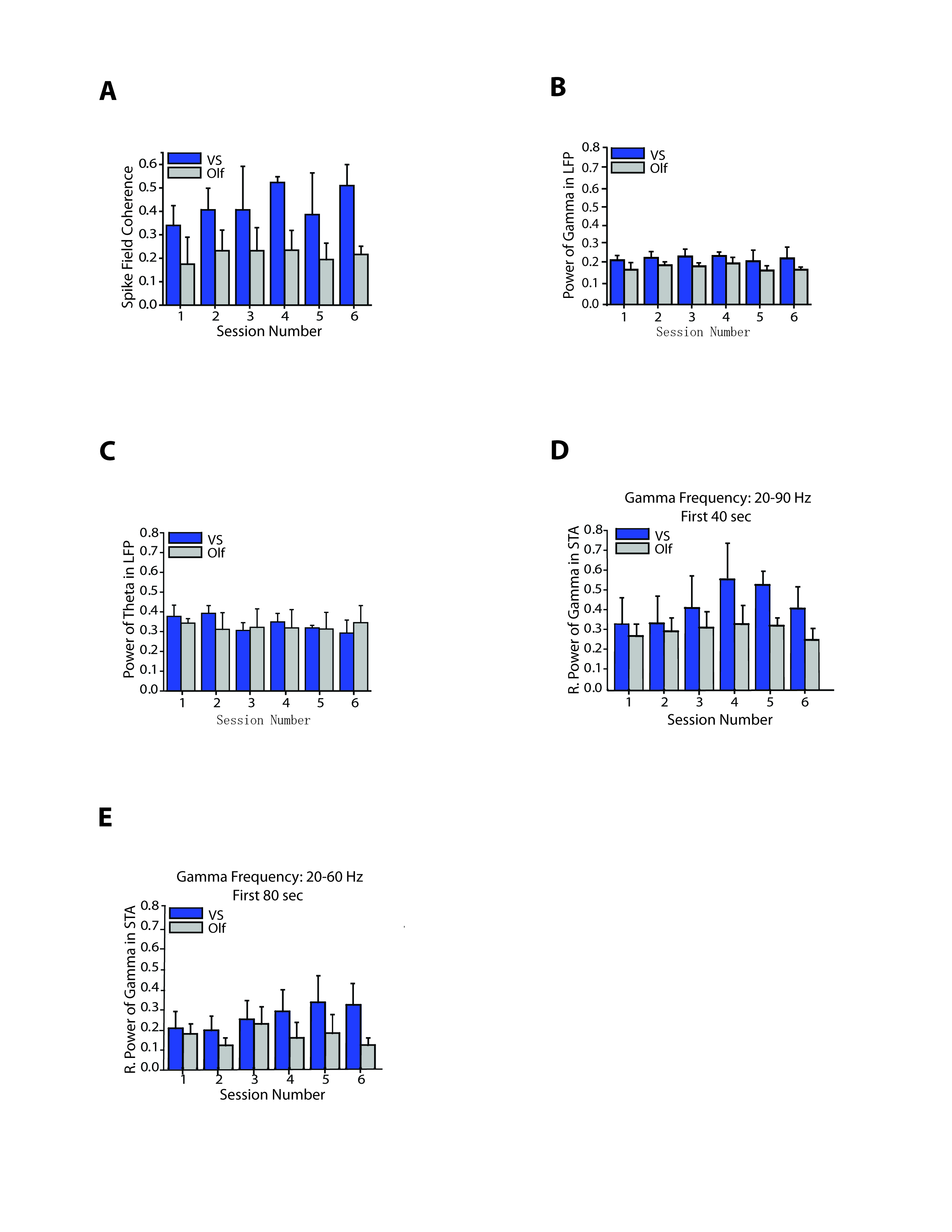

Supplement: Figure S5 — Neuronal synchronicity increases in the visuospatial group without changes in the power of the LFP. (A). Spike field coherence. This form of synchronicity was enhanced in the visuospatial group in comparison to the olfactory group [F(1,23) = 9.24, p<0.03]. However, the effect of session and interaction were not significant [session: F(5,23) = 1.07, p = 0.40; interaction: F(5,23) = 0.35, p = 0.88]. (B and C) The power of gamma (B) and theta (C) in the LFP showed no clear peak at any point during training and no significant differences between the groups. (D) Extending the gamma frequency band (20–90 Hz) showed the same trend observed in the low gamma frequency band (20–60 Hz). However, the differences between the groups were not significant [main effect of groups: F(1,27) = 2.06, p = 0.2; sessions: F(5,27) = 0.90, p = 0.49; interaction: F(5,27) = 0.17, p = 0.97]. (E) Extending the time of analysis to 80 s also showed the same trend observed during the first 40 s, but the differences between the groups were not statistically significant [main effect of groups: F(1,27) = 2.22, p = 0.18; sessions: F(5, 27) = 0.39, p = 0.85; interaction: F(5, 27) = 0.63, p = 0.68]. Olf, olfactory; VS, visuospatial. (1.01 MB DOC) [file pbio.1000140.s005.doc]

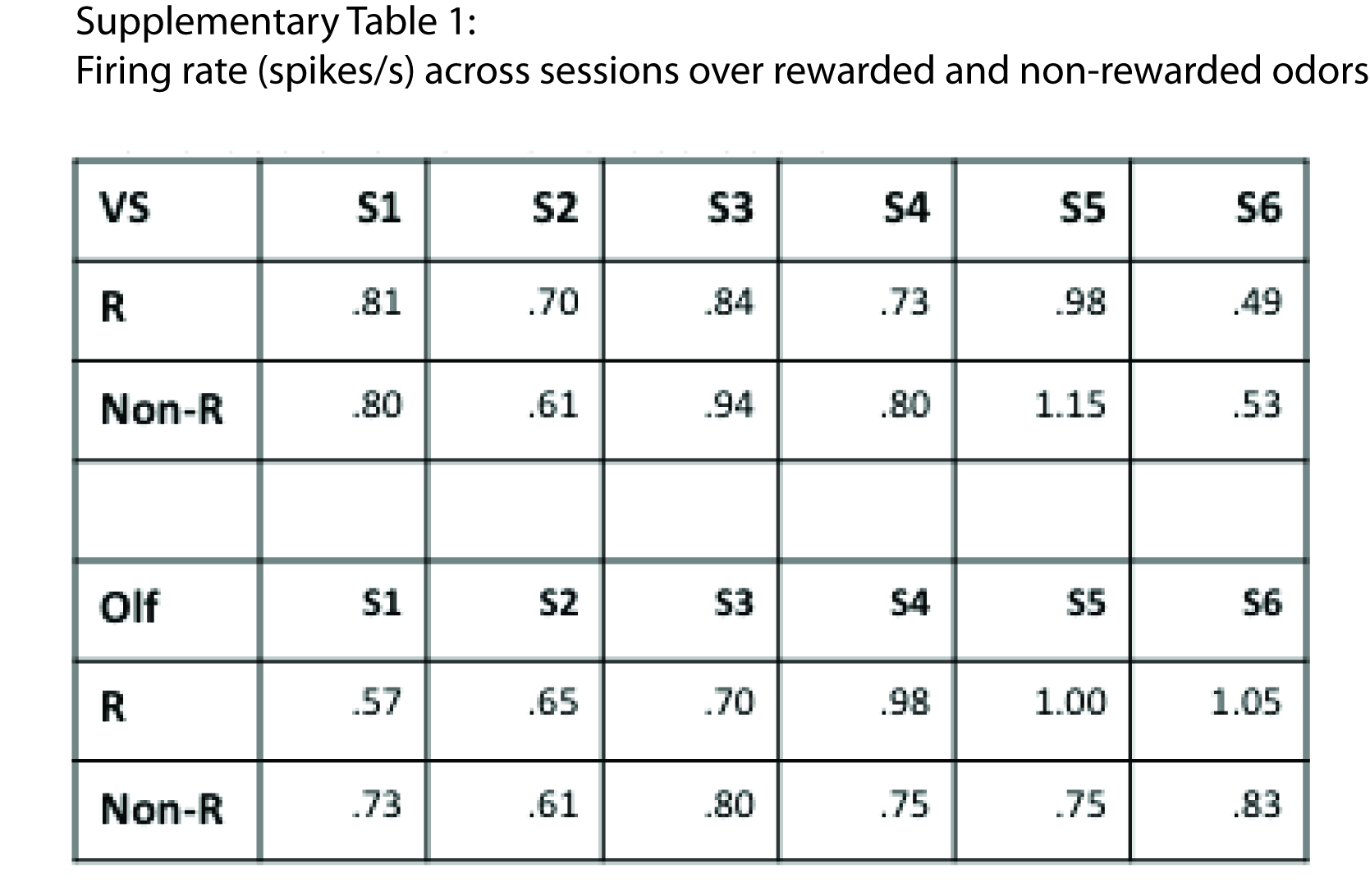

Supplement: Table S1 — Firing rate across sessions (S1 to S6) over the rewarded (R) and nonrewarded (non-R) odors during periods of digging and sniffing (speed threshold below 2 cm/s). (0.55 MB TIF) [file pbio.1000140.s006.tif]

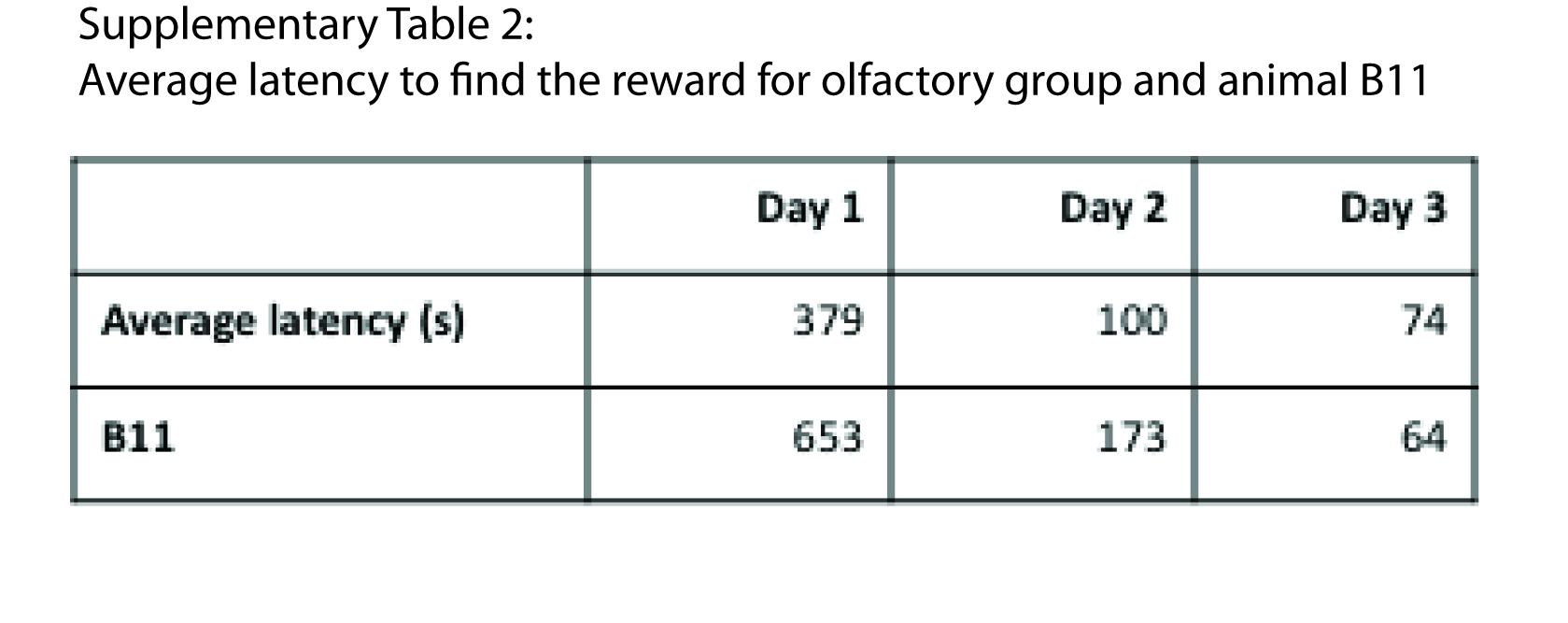

Supplement: Table S2 — Average latency to find the reward across days for the olfactory group and animal B11. The data show that animal B11 latencies were longer than the average on days 1 and 2, which might have contributed to the fact that this was the only animal that displayed significant firing rate responses to a non-rewarded odor in addition to the reward-associated odor. (0.24 MB TIF) [file pbio.1000140.s007.tif]
