# Supplementary figures and images for: Correction: Attention Enhances the Retrieval and Stability of Visuospatial and Olfactory Representations in the Dorsal Hippocampus
Source: PLoS Biol. 2010 Oct 1;8(10):10.1371/annotation/d0e5ef6f-d08b-474a-8fde-aeebeee7369d. doi: 10.1371/annotation/d0e5ef6f-d08b-474a-8fde-aeebeee7369d (PMC2949379; doi:10.1371/annotation/d0e5ef6f-d08b-474a-8fde-aeebeee7369d)

**A**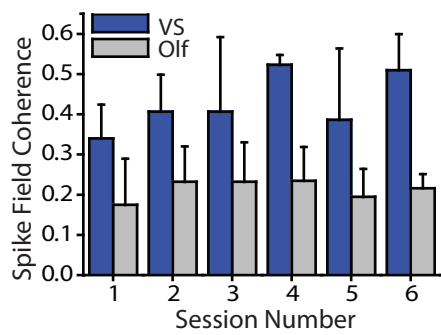**B**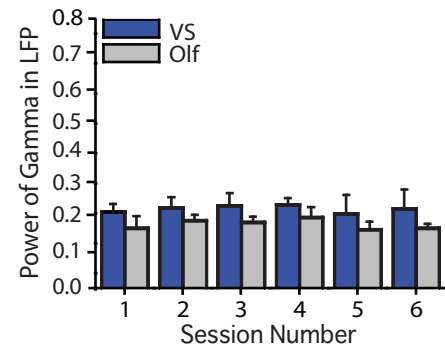**C**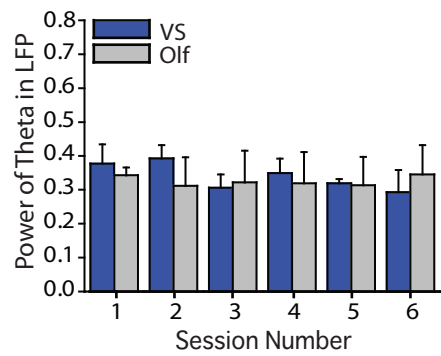**D**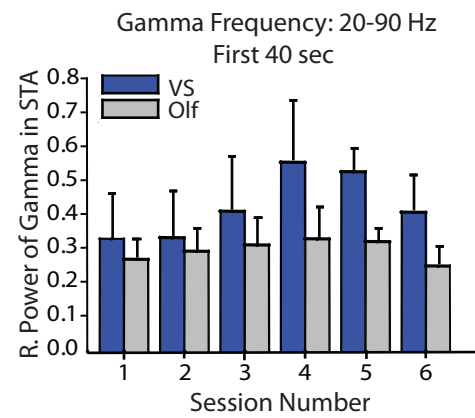**E**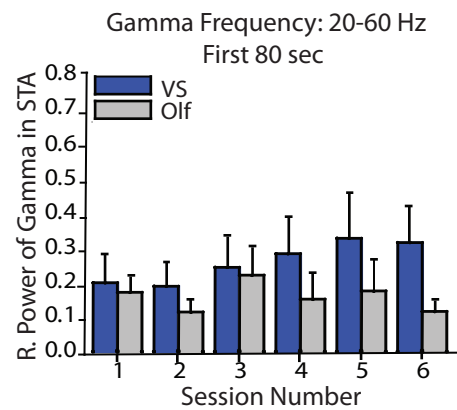

Supplement: Supplementary file 1 [file pbio.d0e5ef6f-d08b-474a-8fde-aeebeee7369d.s001.pdf]
