# Supplementary figures and images for: Correction: Attention Enhances the Retrieval and Stability of Visuospatial and Olfactory Representations in the Dorsal Hippocampus
Source: PLoS Biol. 2010 Oct 1;8(10):10.1371/annotation/5e28240c-6186-43eb-b319-79c391d9468a. doi: 10.1371/annotation/5e28240c-6186-43eb-b319-79c391d9468a (PMC2949380; doi:10.1371/annotation/5e28240c-6186-43eb-b319-79c391d9468a)

## Day 1

Session 1

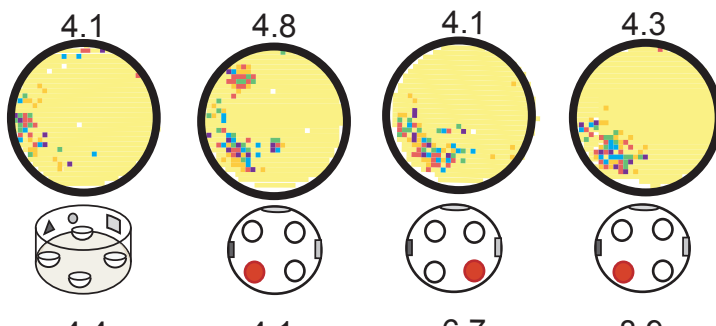

Session 2

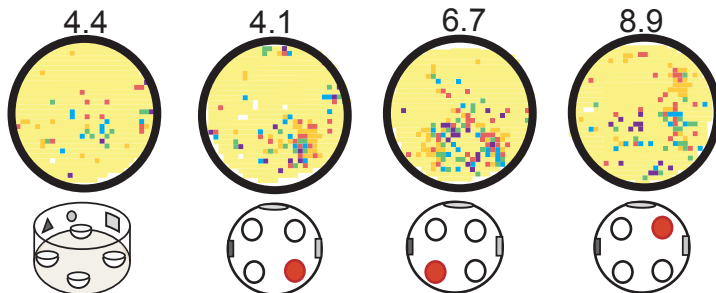

## Day 2

Session 3

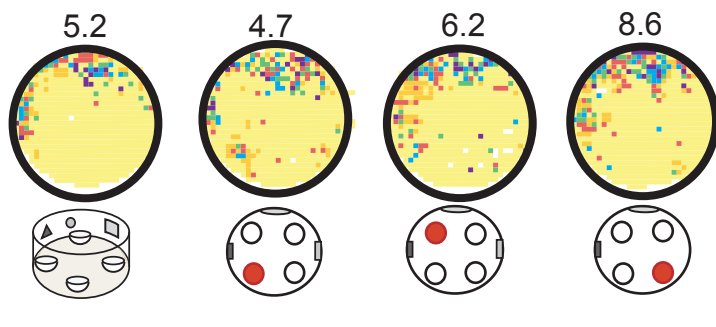

Session 4

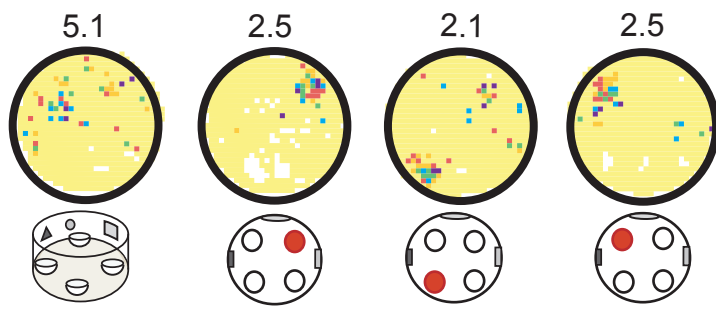

## Day 3

Session 5

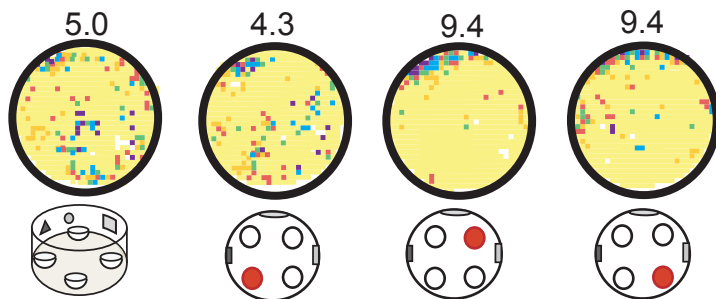

Session 6

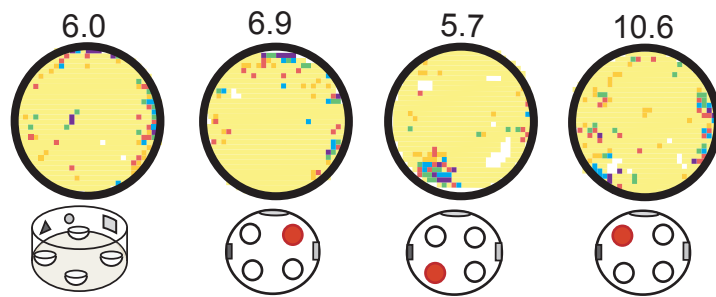

T0 → T1 → T2 → T3

Supporting Figure 4S

Supplement: Supplementary file 1 [file pbio.5e28240c-6186-43eb-b319-79c391d9468a.s001.pdf]
